# Design and Evaluation of a *Zingiber officinale*–Kaolinite–Maltodextrin Delivery System: Antioxidant, Antimicrobial, and Cytotoxic Activity Assessment

**DOI:** 10.3390/pharmaceutics17060751

**Published:** 2025-06-06

**Authors:** Adina-Elena Segneanu, Ionela Amalia Bradu, Gabriela Vlase, Titus Vlase, Cornelia Bejenaru, Ludovic Everard Bejenaru, George Dan Mogoşanu, Maria Viorica Ciocîlteu, Dumitru-Daniel Herea, Eugen Radu Boia

**Affiliations:** 1 Department of Chemistry, Institute for Advanced Environmental Research, West University of Timişoara (ICAM–WUT), 4 Oituz Street, 300086 Timişoara, Romania; adina.segneanu@e-uvt.ro (A.-E.S.); ionela.potinteu@e-uvt.ro (I.A.B.); gabriela.vlase@e-uvt.ro (G.V.); titus.vlase@e-uvt.ro (T.V.); 2 Research Center for Thermal Analyzes in Environmental Problems, West University of Timişoara, 16 Johann Heinrich Pestalozzi Street, 300115 Timişoara, Romania; 3 Drug Research Center, Faculty of Pharmacy, University of Medicine and Pharmacy of Craiova, 2 Petru Rareş Street, 200349 Craiova, Romania; ludovic.bejenaru@umfcv.ro (L.E.B.); george.mogosanu@umfcv.ro (G.D.M.); maria.ciocilteu@umfcv.ro (M.V.C.); 4 Department of Pharmaceutical Botany, Faculty of Pharmacy, University of Medicine and Pharmacy of Craiova, 2 Petru Rareş Street, 200349 Craiova, Romania; 5 Department of Pharmacognosy & Phytotherapy, Faculty of Pharmacy, University of Medicine and Pharmacy of Craiova, 2 Petru Rareş Street, 200349 Craiova, Romania; 6 Department of Instrumental and Analytical Chemistry, Faculty of Pharmacy, University of Medicine and Pharmacy of Craiova, 2 Petru Rareş Street, 200349 Craiova, Romania; 7 National Institute of Research and Development for Technical Physics, 47 Dimitrie Mangeron Avenue, 700050 Iaşi, Romania; dherea@phys-iasi.ro; 8 Department of Ear, Nose, and Throat, Faculty of Medicine, Victor Babeş University of Medicine and Pharmacy Timişoara, 2 Eftimie Murgu Square, 300041 Timişoara, Romania; eugen.boia@umft.ro

**Keywords:** *Zingiber officinale*, kaolinite, micro-spray encapsulation, antioxidant potential, antimicrobial screening, in vitro cytotoxicity

## Abstract

**Background/Objectives**: *Zingiber officinale* Roscoe (*Zingiberaceae*) is widely recognized for its diverse biological activities; however, the stability and bioavailability of its bioactive compounds remain significant challenges. This study aimed to investigate an innovative approach to enhance the stability and efficacy of *Z. officinale* phytoconstituents through advanced encapsulation techniques. **Methods**: Two novel carrier systems were developed: (*i*) direct micro-spray encapsulation of *Z. officinale* in maltodextrin (MZO) and (*ii*) a two-step process involving the creation of a kaolinite-based phytocarrier system (ZO–kaolinite), followed by micro-spray encapsulation in maltodextrin to form the MZO–kaolinite system. **Results**: Comprehensive chemical profiling using GC–MS and ESI–QTOF–MS identified 105 phytochemicals, including terpenoids, gingerols, shogaols, flavonoids, and phenolic acids. Morphostructural analyses (XRD, FTIR, Raman, SEM) confirmed the successful development of the newly engineered kaolinite carrier systems (ZO–kaolinite and MZO–kaolinite systems). Both the ZO–kaolinite and MZO–kaolinite systems exhibited superior antioxidant activity, potent antimicrobial efficacy against major bacterial pathogens (*Staphylococcus aureus*, *Enterococcus faecalis*, *Bacillus cereus*, *Klebsiella pneumoniae*, *Pseudomonas aeruginosa*, *Escherichia coli*), and enhanced cytotoxicity against MCF-7, HCT-116, and HeLa cancer cell lines. **Conclusions**: This study underscores the synergistic action of kaolinite and maltodextrin in developing multifunctional therapeutic systems, emphasizing the importance of phytoconstituent stabilization and nanotechnology in addressing antimicrobial resistance and advancing innovative medical applications.

## 1. Introduction

Natural bioactive compounds have received increasing attention in recent years because of their broad-spectrum therapeutic activities, favorable safety profiles, and cost-effectiveness. Among these, *Zingiber officinale* Roscoe (ginger) stands out as a multifunctional medicinal plant, primarily attributed to its rich and diverse phytochemical composition, including gingerols, shogaols, polyphenols, essential oil, and others [1,2,3]. Numerous studies support the antioxidant, anti-inflammatory, antimicrobial, and anticancer activities of ginger-derived phytoconstituents [1,2,3,4,5,6,7,8,9,10]. Recent research has demonstrated that ginger is efficient in alleviating nausea, gastrointestinal discomfort, osteoarthritic pain, and chemotherapy-induced emesis [1,2,3,4,5,6,7,8].

The anti-inflammatory and antimicrobial potential of ginger has gained heightened relevance amid the growing threat of antimicrobial resistance (AMR), a critical global health concern [7,8,9,10,11,12,13,14]. Ginger bioactive compounds have been shown to inhibit pro-inflammatory cytokine production, disrupt microbial membrane integrity, and suppress pathogenic growth, positioning ginger as a promising natural alternative or adjunct to conventional antimicrobial therapies [15,16,17,18,19,20,21,22].

In parallel, accumulating preclinical data underscore the anticancer potential of ginger’s phytochemicals, particularly gingerols and shogaols, through various molecular mechanisms. These include the induction of apoptosis, inhibition of tumor proliferation, and modulation of key oncogenic signaling pathways, such as nuclear factor-kappa B (NF-κB) and the protein kinase B/mammalian target of rapamycin (Akt/mTOR) axis [10,23,24,25,26]. Collectively, these pharmacological properties highlight the biomedical value of *Z. officinale* as a source of multifunctional therapeutic agents.

Despite these promising properties, the biomedical applications of ginger-based therapeutics remain constrained by several pharmacokinetic challenges, including poor bioavailability, chemical instability, and variability in phytochemical composition [27,28,29,30,31,32,33]. These limitations are exacerbated by the structural complexity of ginger’s secondary metabolites, which significantly impair their absorption, distribution, and therapeutic performance [15,19]. To address these issues, nanocarrier-based drug delivery systems (DDSs) have emerged as a promising strategy to enhance the solubility, improve the chemical stability, and target the delivery of bioactive compounds while simultaneously reducing systemic toxicity and off-target effects [15,19,22,34,35,36].

Among various nanocarriers, clay minerals, particularly kaolinite (Al_2_Si_2_O_5_(OH)_4_), have garnered significant interest due to their inherent biocompatibility, thermal and chemical stability, and notable adsorption capacity [37,38,39]. Kaolinite’s micrometric particle size and substantial specific surface area (~100 m^2^/g) facilitate the efficient loading and controlled release of therapeutic agents, making it a valuable platform for drug delivery applications [39,40,41,42]. Although it exhibits relatively low swelling capacity and limited cation-exchange properties compared to other clays, its layered structure and intercalation potential facilitate the stable incorporation of phytoconstituents, particularly in targeted DDSs for cancer therapy [39,43,44,45].

Recent studies have demonstrated that kaolinite-based systems can enhance the bioavailability of plant-derived compounds and support localized drug delivery, thereby minimizing systemic side effects and improving therapeutic outcomes [39,43,44,45].

In the evolving landscape of natural compound-based therapeutics, recent research efforts have shifted toward designing advanced DDSs with enhanced targeting capabilities, prolonged activity, and greater structural and functional stability [19]. Critical parameters, such as surface chemistry, morphology, thermal stability, and biocompatibility of the carrier, dictate drug loading efficiency, release profiles, and bioactivity [46,47,48].

The development of engineered carrier systems utilizing kaolinite offers a multifunctional strategy that not only improves the solubility and stability of bioactive phytoconstituents but also enables targeted delivery, thereby reducing off-target effects and enhancing therapeutic efficacy [39]. Moreover, such systems can be designed to incorporate controlled-release mechanisms finely tuned to match the pharmacokinetic profiles of herbal compounds, ensuring sustained therapeutic action [39].

In this context, the present study introduces an innovative multifunctional and synergistic tricomponent delivery platform, wherein *Z. officinale* phytoconstituents are first loaded onto kaolinite and subsequently encapsulated within a maltodextrin matrix via spray drying.

To the best of our knowledge, this is the first report describing a kaolinite–maltodextrin microcarrier system specifically engineered for the delivery of *Z. officinale* bioactive compounds. Comprehensive physicochemical characterizations were performed to evaluate the system’s thermal stability, morphology, and the molecular interactions between the phytoconstituents, kaolinite, and maltodextrin. Additionally, the biological performance of the newly prepared system was assessed through in vitro assessments of antioxidant capacity, antimicrobial activity, and cytotoxic potential. This innovative microcarrier platform represents a promising approach to enhance bioavailability, physicochemical stability, and targeted bioactivity of ginger-derived compounds, with potential implications for antimicrobial and anticancer applications.

## 2. Materials and Methods

### 2.1. Chemicals, Reagents, and Plant Material

All reagents utilized in this study were of analytical grade. 2,2-Diphenyl-1-picrylhydrazyl (DPPH), dimethyl sulfoxide (DMSO), potassium persulfate, sodium phosphate, ammonium molybdate, and potassium chloride were sourced from Sigma-Aldrich (München, Germany). A 3-(4,5-dimethylthiazol-2-yl)-2,5-diphenyl tetrazolium bromide (MTT) kit was procured from AAT Bioquest (Pleasanton, CA, USA). Kaolinite and *Z. officinale* (ginger) fresh rhizome were purchased from a local market in Timişoara, Romania. Ultrapure water was used throughout all experimental procedures. Additionally, maltodextrin (with dextrose equivalents ranging from 16.5 to 19.5) was supplied by Carbosynth (Berkshire, UK).

### 2.2. Cell Lines

The cell lines utilized in this study were sourced from the American Type Culture Collection (ATCC) in Manassas, VA, USA. They were cultured at 37 °C in Dulbecco’s Modified Eagle’s Medium (DMEM) from Gibco (Life Technologies, Leicestershire, UK), enriched with 10% fetal bovine serum (FBS) and 1% antibiotic–antimycotic solution from Sigma Aldrich (München, Germany).

### 2.3. Bacterial Strains

This study utilized several bacterial strains sourced from the ATCC (Manassas, VA, USA), including *Staphylococcus aureus* (ATCC 29213), *Bacillus cereus* (ATCC 14579), *Pseudomonas aeruginosa* (ATCC 27853), *Enterococcus faecalis* (ATCC 29212), *Klebsiella pneumoniae* (ATCC 13883), and *Escherichia coli* (ATCC 25922).

### 2.4. Plant Sample Preparation for Chemical Screening

Freeze-dried *Z. officinale* rhizome samples were finely ground using a planetary Fritsch Pulverisette mill (Idar-Oberstein, Germany) at a speed of 780 rpm for 12 min while maintaining a temperature of 23 °C. The resulting powder was then sieved through an American Society for Testing and Materials (ASTM) standard test sieve series to isolate particles in the 0.15 to 0.20 mm range. Following this, the plant material was subjected to sonication extraction with an Elmasonic device (Singen, Germany) for 40 min, at a temperature of 35 °C and a frequency of 75 Hz, using 25 mL of methanol as the solvent. All extracts were prepared in triplicate to ensure accuracy and reproducibility.

### 2.5. GC–MS Analysis

A gas chromatography (GC) analysis was performed using the Shimadzu GCMS-QP2020 NX system, equipped with a ZB-5MS capillary column from Agilent Technologies (Santa Clara, CA, USA). This column measures 50 m in length, has an inner diameter of 0.20 mm, and features a film thickness of 0.33 μm. Helium served as the carrier gas, flowing at a 1 mL/min rate. The oven temperature program commenced at 50 °C, where it was held for 1.5 min before being raised at a rate of 5 °C per minute until reaching a final temperature of 300 °C, which was maintained for an additional three minutes. The injector temperature was set to 270 °C, while the interface temperature was kept at 220 °C. Compounds were ionized at an energy of 80 eV, with data acquisition commencing after a one-minute solvent delay. The mass spectrometer source and MS Quad temperatures were set to 225 °C and 145 °C, respectively. Each analysis was conducted in triplicate to ensure reliability.

Compound identification was achieved by comparing the obtained mass spectra with entries in the National Institute of Standards and Technology (NIST) 2.0 software database (NIST, Gaithersburg, MD, USA) and corroborated through a thorough review of the pertinent literature. To enhance the accuracy of compound identification, retention indices were calculated using the Van den Dool and Kratz formula, employing a C7–C40 *n*-alkane mixture as an internal standard (IS) in the analytical sample. Additionally, Kováts retention indices were determined via logarithmic interpolation, in accordance with established equations for isothermal chromatographic conditions [49,50].

This carefully designed methodology was chosen to guarantee the precise and dependable identification of phytoconstituents. Furthermore, the incorporation of retention indices strengthened the robustness and reproducibility of the results, greatly enhancing confidence in their comparability.

### 2.6. MS Analysis

Mass spectrometry (MS) analyses were performed using an electro-spray ionization–quadrupole time-of-flight–MS (ESI–QTOF–MS) system from Bruker Daltonics (Bremen, Germany). Spectra were recorded in positive ion mode, spanning a mass range of 50 to 3000 *m/z*, with a scan rate of 2.0 scans per second. Collision energies ranged from 20 to 80 eV, and the source block temperature was consistently maintained at 80 °C. The identification of phytoconstituents was achieved by referencing the NIST/National Bureau of Standards (NBS)-3 library (NIST, Gaithersburg, MD, USA) and by comparing the results with a thorough review of the pertinent scientific literature.

### 2.7. Spray-Drying Process

The spray-drying process was conducted using a Mini Spray Dryer B-290. The operational parameters were optimized, establishing a feed flow rate of 8 mL/min. The inlet and outlet temperatures were held at 125 °C and 70 °C, respectively. An airflow rate of 35 m^3^/h was employed, along with a compressor air pressure of 0.05 MPa and a nozzle diameter of 0.7 mm. The system also functioned with 100% suction airflow in an environment with approximately 80% relative humidity [51,52].

### 2.8. Phytocarrier (ZO–Kaolinite) System Preparation

The *Z. officinale* (ZO)–kaolinite system was prepared by mixing *Z. officinale* (solid herb rhizome samples, prepared as previously described) with kaolinite in a 1:1 mass ratio. This mixture was ground and homogenized for 18 min using a planetary Fritsch Pulverisette mill (Idar-Oberstein, Germany), at a speed of 780 rpm, while maintaining a temperature of 22 °C. Each experiment was conducted in triplicate.

### 2.9. Preparation of the Maltodextrin–ZO (MZO) Carrier

To prepare the MZO carrier, a mixture containing 2.0 g of dried *Z. officinale* and 2.0 g of maltodextrin was dissolved in 50 mL of ultrapure water and thoroughly homogenized. The resulting solution was then incubated at 35 °C for 25 min while continuously stirred. After incubation, the mixture was centrifuged for 10 min and filtered through Whatman filter paper (0.45 μm). The filtered solution was subsequently processed using a spray dryer, and the resulting dried powder was carefully collected and stored in an opaque, airtight container at 23 °C for future analysis. All experiments were conducted in triplicate.

### 2.10. Preparation of the Maltodextrin–ZO–Kaolinite (MZO–Kaolinite) System

Using the same experimental procedure, the MZO–kaolinite system was developed by combining the ZO–kaolinite system with maltodextrin in a 1:1 mass ratio. All experiments were conducted in triplicate.

### 2.11. Characterization of Carrier Systems

#### 2.11.1. FTIR Spectroscopy

Fourier transform infrared (FTIR) data were obtained using a Shimadzu AIM-9000 spectrometer fitted with attenuated total reflectance (ATR) accessories (Shimadzu, Tokyo, Japan). The spectra were captured over 20 scans at a resolution of 4 cm^−1^ within the range of 4000 to 400 cm^−1^. Wavelength assignments were determined through a thorough analysis of the pertinent literature.

#### 2.11.2. XRD Analysis

X-ray diffraction (XRD) analysis was performed using a Bruker AXS D8-Advance X-ray diffractometer (Bruker AXS GmbH, Karlsruhe, Germany), which utilized CuKα radiation (λ = 0.1541 nm). The instrument was equipped with a rotating sample stage, an Anton Paar TTK low-temperature cell (operating between −180 °C and 450 °C), a high vacuum system, inert atmosphere capabilities, relative humidity control, and an Anton Paar TTK high-temperature cell (up to 1600 °C). The XRD patterns obtained were systematically analyzed and compared with reference data from the International Centre for Diffraction Data (ICDD) Powder Diffraction Database (ICDD file 04-015-9120). Additionally, the whole powder profile fitting (WPPF) methodology was employed to determine the average crystallite size and phase composition of the samples.

#### 2.11.3. SEM Analysis

Scanning electron microscopy (SEM) analysis was performed using a JSM-IT200 InTouchScope™ scanning electron microscope (Freising, Germany) integrated with an energy-dispersive X-ray (EDX) spectroscopy (EDS) system. The instrument was equipped with a field emission gun (FEG), ensuring high-resolution imaging and precise elemental analysis.

#### 2.11.4. DLS Particle Size Distribution Analysis

Dynamic light scattering (DLS) analysis was conducted using a Microtrac/Nanotrac 252 (Montgomeryville, PA, USA). Measurements were performed at room temperature (RT; 23 °C), with a scattering angle of 172°. Each sample was analyzed in triplicate.

#### 2.11.5. Encapsulation Efficiency, Loading Capacity, and Encapsulation Yield

The encapsulation efficiency (EE%) was determined as the percentage of the total mass (g) of the *Z. officinale* sample and the ZO–kaolinite system relative to the total mass (g) of raw materials used in the encapsulation process, following Equation (1) [51,52,53,54]:

(1) $$EE\;\left(\%\right)=\frac{amount\;of\;compound\;encapsulated\;(g)}{amount\;compound\;used\;as\;raw\;materials\;(g)}\times 100$$

The loading capacity (EC%) was determined as the ratio of the capsule weight, excluding the mass of the raw material compounds (g), to the total mass of the raw material compounds (g), as described by Equation (2) [51,52,54]:

(2) $$EC\;\left(\%\right)=\frac{weight\;of\;capsules\;(g)}{weigh\;of\;compound\;used\;as\;raw\;material+weigt\;of\;maldextrin\;(g)}\times 100$$

The encapsulation yield (EY%) was calculated as the ratio of the microencapsulated material within maltodextrin to the total weight of raw materials, as defined by Equation (3) [53,54]:

(3) $$EY\;\left(\%\right)=\frac{weight\;of\;capsules-amount\;of\;compound\;used\;as\;raw\;material\;(g)}{amount\;compound\;used\;as\;raw\;materials\;(g)}\times 100$$

The compound content and EE% of maltodextrin microparticles were quantified using a Perkin-Elmer Lambda 35 ultraviolet–visible (UV–Vis) spectrophotometer (Perkin Elmer, Waltham, MA, USA). All absorbance measurements were performed at RT in a 10 mm UV–Vis spectroscopy cell, with an ethanol–chloroform (1:1, *v/v*) solution as the blank. For sample preparation, 20 mg of the encapsulated material was subjected to ultrasound-assisted extraction (UAE) at a frequency of 70 kHz in 25 mL of a solvent mixture (hydrochloric acid–ethanol–chloroform, 3:2:2, *v/v/v*) for 45 min at 22 °C. Following extraction, the samples were centrifuged, and the supernatant concentration was determined using atomic UV–Vis spectroscopy. Each experiment was performed in triplicate [53,54].

### 2.12. Thermal Analysis

The thermal stability of the samples was assessed using a Mettler Toledo TG/DSC3+ thermal analyzer equipped with a thermogravimetry/differential thermal analysis (TG/DTA) high-temperature (HT) sensor. The thermal behavior was studied over a temperature range of 25–400 °C, under a synthetic air atmosphere (5.0), with all analyses conducted in 40 μL aluminum crucibles.

### 2.13. Estimation of Total Phenolic Content and Antioxidant Activity

The total phenolic content (TPC) of *Z. officinale*, the ZO–kaolinite system, the MZO carrier, and the MZO–kaolinite system was quantified using the Folin–Ciocalteu assay. To evaluate the antioxidant activity of these samples, we employed several methods, including the ferric reducing antioxidant power (FRAP) assay, the DPPH radical scavenging assay, and the phosphomolybdate assay, to determine total antioxidant capacity. All antioxidant activity experiments were conducted in triplicate to ensure reproducibility.

#### 2.13.1. Sample Preparation Procedure

During the sample preparation procedure, a 0.40 g aliquot of the *Z. officinale* sample was mixed with 10 mL of 70% ethanol and stirred continuously for 10 h at RT (23 °C) to ensure thorough extraction. Following this, the mixture was centrifuged at 5000 rpm for 15 min to separate the solid residues, and the resulting supernatant was carefully collected for subsequent assessment of its antioxidant potential. The same experimental protocol was conducted for the ZO–kaolinite system, the MZO carrier, and the MZO–kaolinite system.

#### 2.13.2. TPC Assay

The TPC of *Z. officinale*, the ZO–kaolinite system, the MZO carrier, and the MZO–kaolinite system was quantified spectrophotometrically using a FLUOstar Optima UV–Vis spectrometer (BMG Labtech, Offenburg, Germany), following the protocol described in our previously published papers [53,54,55,56]. The results were reported in terms of mg gallic acid equivalents (GAEs)/g sample. The sample concentrations were determined based on the linear Equation (4) derived from the standard calibration curve, which exhibited a strong correlation coefficient (*R*^2^ = 0.9867):

*y* = 0.0022*x* + 0.0225 (4)

#### 2.13.3. FRAP Assay

The FRAP antioxidant activity of *Z. officinale*, the ZO–kaolinite system, the MZO carrier, and the MZO–kaolinite system were evaluated spectrophotometrically at 595 nm using a FLUOstar Optima UV–Vis spectrometer (BMG Labtech) and a FRAP assay kit, following the methodology detailed in our previous publications [55,56,57]. The antioxidant activity was expressed in millimolar (mM) Fe^2+^ equivalents, calculated using Equation (5):

(5) $$\mathrm{FRAP}=\frac{C_{{Fe}^{2+}}\times F}{V}$$

where *C_Fe_*^2+^ represents the concentration (nM) of iron (Fe^2+^) ions generated from the calibration curve for each sample, *F* denotes the dilution factor, and *V* refers to the sample volume (μL).

Sample concentrations were determined based on the linear Equation (6) derived from the standard calibration curve, which demonstrated a high correlation coefficient (*R*^2^ = 0.9998):

*y* = 0.0006*x* + 0.0909 (6)

#### 2.13.4. DPPH Radical Scavenging Assay

The DPPH radical scavenging activity of *Z. officinale*, the ZO–kaolinite system, the MZO carrier, and the MZO–kaolinite system samples were assessed following the methodology outlined in our previous publications [53,54,55,56]. Absorbance measurements were taken at 520 nm using a FLUOstar Optima UV–Vis spectrometer (BMG Labtech). The half-maximal inhibitory concentration (IC_50_) values (μg/mL) were calculated based on the percentage of inhibition, Inh (%), determined from the calibration curve for each sample using Equation (7):

(7) $$\mathrm{Inh}(\%)=\frac{\left(A_{0}-A_{1}\right)}{A_{0}}\times 100$$

where *A*_0_ represents the absorbance of the control and *A*_1_ denotes the absorbance of the sample.

#### 2.13.5. Phosphomolybdate Assay (Total Antioxidant Capacity)

The total antioxidant capacity (TAC) assay of *Z. officinale*, the ZO–kaolinite system, the MZO carrier, and the MZO–kaolinite system samples was carried out by the phosphomolybdenum procedure, according to the methodology described in our previous paper, using ascorbic acid as standard [58]. The absorbance was measured at 765 nm using a UV–Vis Perkin-Elmer Lambda 35 (Perkin Elmer, Waltham, MA, USA). TAC (%) was determined according to Equation (8):

(8) $$\mathrm{TAC}(\%)=\frac{\left(A_{0}-A_{1}\right)}{A_{0}}\times 100$$

where *A*_0_ represents the absorbance of the control and *A*_1_ denotes the absorbance of the sample.

The results are expressed in μg/mL of ascorbic acid equivalents (AAEs).

### 2.14. Antimicrobial Activity

The antimicrobial activity of *Z. officinale*, the ZO–kaolinite system, the MZO carrier, and the MZO–kaolinite system was assessed using a combination of agar well diffusion assays, minimum inhibitory concentrations (MICs), and minimum bactericidal concentrations (MBCs). MIC and MBC values were determined via the microbroth dilution method using Müller–Hinton medium. The MIC was determined as the lowest concentration of the test compound that successfully inhibited bacterial growth, while the MBC corresponded to the lowest concentration at which no visible bacterial growth was observed after 14 h of incubation.

Microbial growth inhibition was quantified by measuring optical density at 600 nm with a T90+ UV–Vis spectrophotometer (PG Instruments, Lutterworth, UK) [59]. Nutrient agar and nutrient broth were prepared according to the manufacturer’s instructions and sterilized via autoclaving at 125 °C for 30 min. The microbial suspension was adjusted to the 0.5 McFarland Standard, corresponding to 1.5 × 10^8^ colony-forming units per milliliter (CFU/mL). All experiments were conducted in triplicate to ensure reliability. Five serial dilutions (100, 125, 150, 175, and 200 μg/mL) of each sample were prepared in 25% DMSO [54,55,59]. The antimicrobial potential was further evaluated using the agar well diffusion method, following the protocol established in our previous study [54,55].

Each experiment was performed in triplicate to enhance statistical accuracy and reproducibility [60,61,62].

### 2.15. Cell Culture Procedure

#### 2.15.1. Cell Culture and Treatment

This study utilized MCF-7 (breast cancer), HCT-116 (colorectal cancer), and HeLa (cervical cancer) cell lines, all sourced from ATCC (Manassas, VA, USA). The cells were cultured in DMEM supplemented with FBS and a 1% antibiotic–antimycotic solution, maintained at 37 °C with 5% CO_2_ and 100% humidity. The cells were seeded at a density of 4 × 10^3^ cells per well in 96-well plates and incubated 24 h to reach approximately 90% confluency. The medium was then replaced with fresh culture medium containing varying concentrations (75, 100, 125, 150, 175, and 200 μg/mL) of *Z. officinale*, the ZO–kaolinite system, the MZO carrier, and the MZO–kaolinite system [54,55,56]. A control group, consisting of standard fresh medium, along with positive and negative controls, was included. Each experimental condition was performed in triplicate. Following a 24 h incubation period at 37 °C under 5% CO_2_, cell viability was assessed.

#### 2.15.2. MTT Assay

Cell viability was evaluated using the MTT assay. After the incubation period, the culture medium was carefully aspirated from each well, and 25 μL of MTT reagent was added to each well. The plates were incubated for an additional two hours at 37 °C in a CO_2_ incubator to allow formazan crystal formation. Subsequently, the formed formazan crystals were solubilized with DMSO, and the absorbance was measured at 540 nm using a Multi-Mode Microplate Reader Synergy HTX spectrophotometer (Agilent Technologies, Santa Clara, CA, USA). Cell viability, CV (%), was determined using Equation (9):

(9) $$\mathrm{CV}(\%)=\frac{\left({OD}_{sample}-{OD}_{blank}\right)}{\left({OD}_{control}-{OD}_{blank}\right)}\times 100$$

where *OD_sample_* represents the optical density of wells containing cells treated with the test sample, *OD_control_* corresponds to wells with untreated cells, and *OD_blank_* refers to the optical density of the cell culture medium without cells.

The positive control included untreated cells, MTT solution, and DMSO, whereas the negative control consisted of non-viable (dead) cells exposed to MTT solution and DMSO. The IC_50_ was determined by plotting cell viability percentages against the tested concentrations (75, 100, 125, 150, 175, and 200 μg/mL) [54,55,56,63]. IC_50_ was defined as the concentration at which 50% of the cells remained viable in the MCF-7, HCT-116, and HeLa cell lines. The data were plotted, and IC_50_ values were calculated as a result [54,55,56].

### 2.16. Statistical Analysis

All experiments, including sample measurements, calibration curves, and concentration determinations, were performed in triplicate. The results are expressed as mean ± standard deviation (SD). Statistical analyses were conducted using Student’s *t*-test, utilizing Microsoft Office Excel 2019 (Microsoft Corporation, Redmond, WA, USA). For multiple comparisons, Dunnett’s post hoc test was employed following a one-way analysis of variance (ANOVA). A *p*-value of less than 0.05 was deemed statistically significant.

## 3. Results

### 3.1. GC–MS Analysis

**Figure 1:**
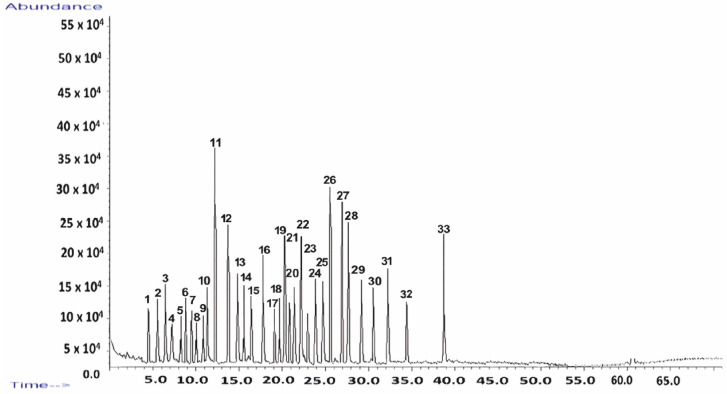
Total ion chromatogram of the *Z. officinale* sample.

GC–MS was employed to perform a comprehensive molecular characterization of *Z. officinale*, enabling the identification of key bioactive constituents, including terpenes, gingerols, and shogaols, which play a crucial role in the plant’s therapeutic properties. This method was chosen for its high sensitivity and precision in detecting both volatile and semi-volatile compounds. The GC–MS analysis not only provided a detailed chemical profile but also delivered valuable insights into the stability of these compounds, their potential interactions with the kaolinite-based carrier system, and their implications for controlled release kinetics. The chemical profile of *Z. officinale* was systematically analyzed, allowing for the efficient separation and identification of bioactive compounds. The resulting chromatographic fingerprint is presented in Figure 1, with a detailed breakdown of the identified compounds, including their retention times and relative abundances, provided in Table 1. These findings confirm the complex phytochemical composition of *Z. officinale*, underscoring its pharmacological relevance, and are fully detailed in the accompanying Figure 1 and Table 1.

The GC–MS analysis revealed a total of 33 compounds, collectively constituting 90.31% of the total peak area in *Z. officinale* (Figure 1).

### 3.2. MS Analysis

The selection of this analytical method was driven by the necessity for thorough molecular characterization to facilitate the development of the engineered kaolinite-based carrier system. By identifying key bioactive constituents, this analysis provides essential insights into their chemical stability, potential interactions with the kaolinite matrix, and their implications for controlled release kinetics. Furthermore, the identification of bioactive phytoconstituents highlights the significance of the newly engineered carrier systems, reinforcing their potential for biomedical applications.

**Figure 2:**
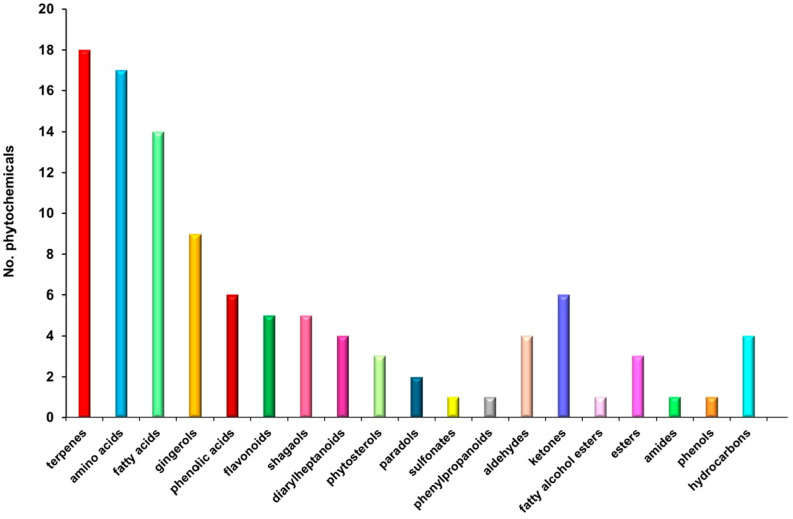
Phytoconstituent classification bar chart of *Z. officinale*.

The MS analysis of *Z. officinale* revealed a diverse array of bioactive compounds, including amino acids, gingerols, shogaols, terpenes, fatty acids, flavonoids, phenolic acids, phytosterols, paradols, hydrocarbons, esters, phenylpropanoids, diarylheptanoids, fatty alcohols, aldehydes, and ketones (Figure 2; Table 2). These results corroborate previous studies, reaffirming the complex phytochemical composition of *Z. officinale* and its broad-spectrum therapeutic potential.

### 3.3. Chemical Screening

A comprehensive chemical profiling of the *Z. officinale* sample was conducted using GC–MS and ESI–QTOF–MS to identify its bioactive constituents. The GC–MS analysis specifically revealed the presence of volatile and semi-volatile compounds (Figure 2; Table 2), while the ESI–QTOF–MS analysis led to the identification of 105 distinct phytoconstituents. These bioactive molecules encompass a broad spectrum of chemical classes, including terpenoids (17.14%), amino acids (16.19%), fatty acids (13.34%), gingerols (9.52%), shogaols (3.81%), paradols (1.90%), phenolic acids (5.71%), flavonoids (4.76%), phytosterols (2.85%), diarylheptanoids (3.80%), esters (2.86%), hydrocarbons (3.81%), fatty alcohols ester, aldehydes and ketones (9.52%), phenylpropanoids (0.95%), sulfonates (0.95%), phenols (0.95%), and amides (0.95%). The distribution and relative abundance of these biomolecules, as determined through MS analysis, are presented in Figure 2, while a comprehensive list of the identified compounds is provided in Table 2.

### 3.4. Key Aroma-Active Compounds and Their Contribution to the Flavor Profile

**Figure 3:**
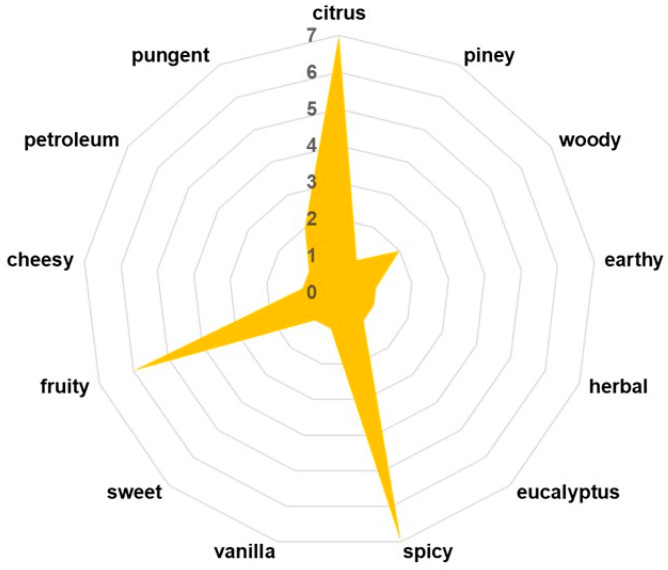
The VOC odor profile of the compounds found in the *Z. officinale* sample. VOC: volatile organic compound.

The sensory profile of *Z. officinale* is primarily defined by its volatile organic compounds (VOCs), which not only contribute to its characteristic aroma and flavor but also play a crucial role in its pharmacological properties, including antimicrobial, anti-inflammatory, and digestive benefits [10]. In this study, the development of phyto-engineered kaolinite-based carrier systems necessitates a comprehensive evaluation of *Z. officinale*’s VOC composition to assess potential interactions with kaolinite particles. The maltodextrin encapsulation process is designed to preserve and regulate the release of these bioactive volatiles, ensuring sustained therapeutic efficacy while maintaining ginger’s sensory attributes. Therefore, identifying key volatile constituents is essential for understanding the functional stability and sensory integrity of the ZO–kaolinite systems before and after encapsulation in the biopolymeric matrix. A detailed VOC profile is presented in Table 3 and Figure 3, highlighting the major constituents responsible for ginger’s distinctive sensory complexity.

### 3.5. Phytocarrier System

#### 3.5.1. FTIR Analysis

FTIR spectroscopy was employed to elucidate the complex chemical interactions between kaolinite and various phytochemicals derived from *Z. officinale*, providing critical confirmation of the formation of the ZO–kaolinite system, the MZO carrier, and the MZO–kaolinite system.

**Figure 4:**
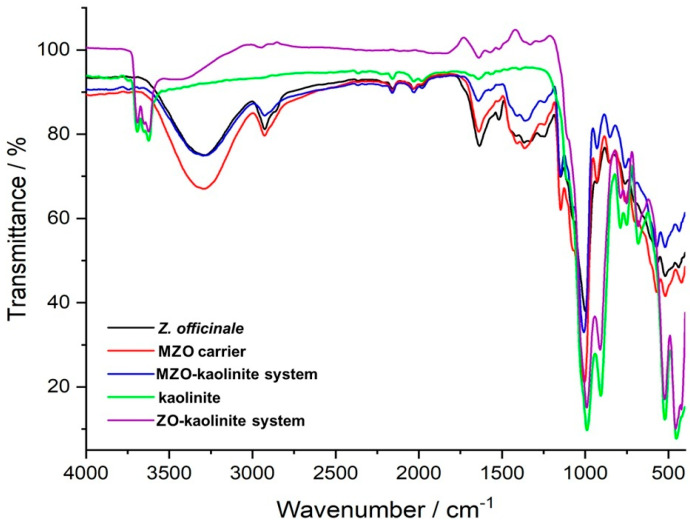
FTIR spectra of *Z. officinale*, kaolinite, the ZO–kaolinite system, the MZO carrier, and the MZO–kaolinite system. FTIR: Fourier transform infrared spectroscopy; ZO: *Z. officinale*; ZO–kaolinite: *Z. officinale*–kaolinite system; MZO carrier: maltodextrin–*Z. officinale* carrier; MZO–kaolinite: Maltodextrin–*Z. officinale*–kaolinite system.

The FTIR spectrum of the *Z. officinale* sample (Figure 4, black line) revealed a diverse spectrum of biomolecular classes, including terpenoids, amino acids, gingerols, fatty acids, phenolic acids, flavonoids, phytosterols, diarylheptanoids, alkaloids, phytosterols, fatty alcohol esters, phenylpropanoids, shogaols, paradols, and various other compounds (Table 4). This extensive compositional diversity underscores the intricate chemical landscape of the ginger sample.

Table 4 displays the FTIR spectrum of the ginger sample, highlighting a variety of peaks that correspond to the functional groups and molecular vibrations characteristic of the compounds present in the ginger.

The FTIR spectrum of the ZO–kaolinite system (Figure 4, purple line) reveals distinct vibrational bands corresponding to its structural components, specifically the characteristic peaks associated with *Z. officinale* phytoconstituents (Figure 4, black line; Table 4) and kaolinite (Figure 4, green line). The major absorption bands of *Z. officinale* are observed at 3296 cm^−1^ (O–H stretching), 2923 cm^−1^ (asymmetric –CH_2_ vibration), 2854 cm^−1^ (asymmetric and symmetric CH stretching), and 1711 cm^−1^ (C=O stretching vibration). Additionally, functional groups linked to key bioactive compounds are identified, including coumarins (1607 cm^−1^, C=C), flavonoids (1637 cm^−1^, C=O), alkaloids (1602 cm^−1^, C=C and N–H), amides (1518 cm^−1^, C=O and N–O stretching), amines (1240 cm^−1^, C–N; 1036 cm^−1^, N–H), carbohydrates (1003 cm^−1^, C–O), and aromatic rings (875 cm^−1^, C–H bending) [82,83]. Similarly, the characteristic absorption bands of kaolinite (Figure 4, green line) are observed at 3686, 3652, 3619, and 3552 cm^−1^ (O–H stretching), 1117, 1066, 980, 912, and 740 cm^−1^ (Si–O stretching), and 683 cm^−1^ (Al–OH vibrations) [74]. The shifts and increased intensities in the O–H, N–H, C–O, and C–H absorption bands in the ZO–kaolinite system suggest strong hydrogen bonding and electrostatic interactions, confirming the successful preparation of this newly engineered carrier system. Furthermore, the retention of kaolinite’s key structural features within the ZO–kaolinite composite supports its structural stability and functionality.

The FTIR spectrum of the MZO carrier (Figure 4, red line) highlights the successful encapsulation of *Z. officinale* within maltodextrin, as evidenced by characteristic carbohydrate vibrational bands at 1024 cm^−1^ and 1152 cm^−1^ (C–O–C stretching), 926 cm^−1^ (α-1,4 glycosidic bond), 848 cm^−1^ (C–H bending), and 759 cm^−1^ (pyranose ring vibration) [54,75,76]. These spectral features, alongside the retention of distinct peaks corresponding to ginger phytochemicals, confirm the molecular integrity and stability of the encapsulated bioactive compounds.

The MZO–kaolinite system (Figure 4, blue line) further demonstrates the effective integration of maltodextrin, kaolinite, and ginger phytochemicals. This is evidenced by the superimposition of vibrational bands, shifts in –OH and N–H absorption bands indicative of enhanced hydrogen bonding, and the emergence of new peaks at 1427 cm^−1^ and 878 cm^−1^, confirming successful encapsulation of the ZO–kaolinite system within the maltodextrin matrix. Overall, FTIR spectroscopic analysis provides compelling evidence for the successful development of the novel ZO–kaolinite system. The distinctive maltodextrin absorption bands observed in the MZO and MZO–kaolinite systems underscore the protective role of the biopolymeric matrix in stabilizing and preserving the encapsulated bioactive compounds. Additionally, peak shifts, intensity variations, and the emergence of new absorption bands indicate strong molecular interactions among ginger phytochemicals, kaolinite, and maltodextrin, resulting in well-integrated and structurally stable encapsulated systems.

#### 3.5.2. XRD Analysis

**Figure 5:**
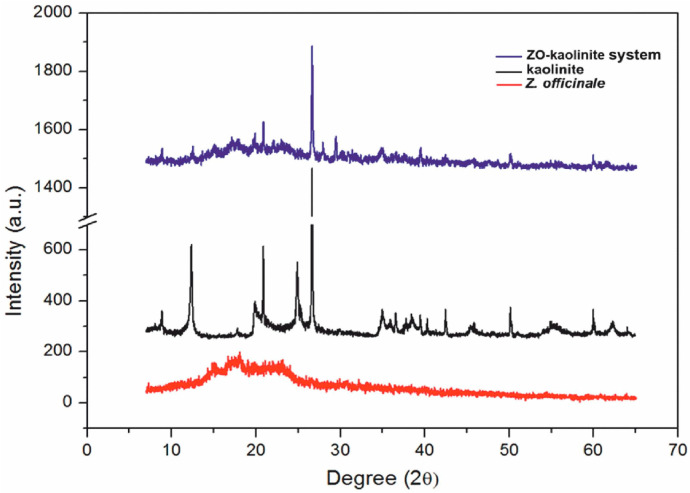
XRD patterns of the *Z. officinale* sample (red line), kaolinite (black line), and the ZO–kaolinite system (blue line). XRD: X-ray diffraction.

The XRD patterns (Figure 5) reveal distinct crystalline and amorphous characteristics, providing insights into their structural properties and material interactions.

The XRD pattern of the *Z. officinale* sample (Figure 5, red line) exhibits a broad hump in the 10–30° (2θ) range, characteristic of an amorphous or semi-crystalline organic structure. The absence of sharp diffraction peaks suggests that *Z. officinale* is primarily composed of biopolymers, starch, and phytochemicals, which lack a well-ordered crystalline lattice [53,54,55,56,57,77]. This broad diffraction feature confirms its organic, fibrous nature, which contributes to its porosity and structural irregularity. Specifically, the diffraction peak observed at approximately 15–20° (2θ) suggests the presence of cellulose and other phytoconstituents, including terpenoids, alkaloids, and flavonoids. Terpenoids typically exhibit characteristic diffraction peaks within the 10–20° (2θ) range, reflecting their molecular structure and crystallinity. Alkaloids, in contrast, are identified by distinct peaks at approximately 14.5° and 18.5° (2θ), indicative of nitrogen-containing bioactive compounds. Flavonoids are predominantly detected within the 20–30° (2θ) range, where their crystalline forms exhibit characteristic reflections [54]. Additionally, the weak, broad peaks near 20–22° (2θ) confirm the presence of semi-crystalline polysaccharides within the *Z. officinale* matrix [84].

The XRD pattern of kaolinite (Figure 5, black line) displays several sharp and well-defined peaks, particularly in the 20–30° (2θ) region, corresponding to the crystalline aluminosilicate phase characteristic of kaolinite. These peaks confirm its highly ordered layered structure and the presence of key kaolinite mineral phases. The primary diffraction peaks at 12.4°, 20.4°, 24.9°, 35°, and 51° (2θ) are indicative of a triclinic kaolinite structure. The sharpness and intensity of these peaks suggest high crystallinity, a common feature of clay minerals, which enhances their structural stability and adsorption capacity [58,78].

The ZO–kaolinite system (Figure 5, blue line) exhibits an intermediate XRD pattern, combining features from both kaolinite and *Z. officinale*. While the sharp peaks of kaolinite remain visible, their relative intensities are reduced, suggesting the intercalation of organic molecules within the kaolinite layers. Additionally, the broad amorphous hump from *Z. officinale* remains present, indicating a partial interaction between kaolinite and *Z. officinale* bioactive compounds, likely through adsorption or intercalation [79]. The slight broadening and reduced intensity of kaolinite peaks, along with a shift toward lower 2θ angles, suggest an increase in interlayer spacing, further supporting the hypothesis of organic molecule intercalation [79]. The presence of a broader amorphous region in the 15–25° (2θ) range confirms the dispersion of *Z. officinale* phytoconstituents within the kaolinite structure. Moreover, the emergence of new peaks in the 8–10° (2θ) range suggests the formation of a hybrid intercalated phase, reinforcing the idea of strong molecular interactions between kaolinite and *Z. officinale* bioactive compounds. Overall, the ZO–kaolinite system exhibits both organic (amorphous) and inorganic (crystalline) characteristics, confirming the successful integration of *Z. officinale* into the kaolinite framework. This hybrid structure suggests potential functional enhancements, such as improved stability, adsorption properties, and controlled release of bioactive compounds, compared to the individual components.

#### 3.5.3. SEM–EDX Analysis

**Figure 6:**
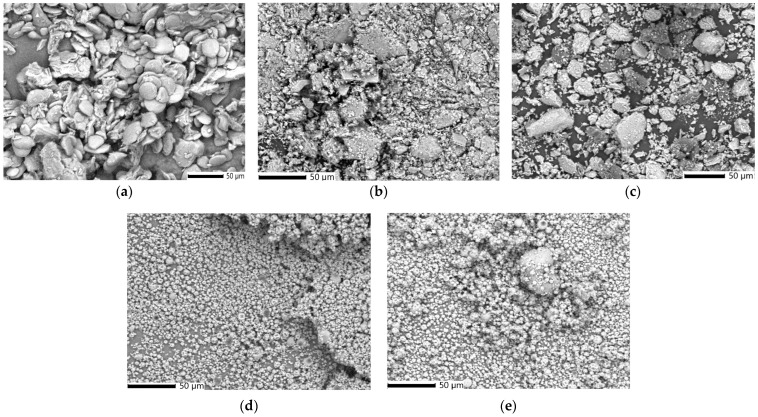
SEM micrograph of the *Z. officinale* sample (**a**), kaolinite (**b**), ZO–kaolinite system (**c**), MZO carrier (**d**), and MZO–kaolinite system (**e**). SEM: scanning electron microscopy.

Figure 6a–d displays SEM micrographs, revealing the qualitative morphological characteristics of the *Z. officinale* sample and the ZO–kaolinite system before and after encapsulation within the biopolymeric matrix.

The SEM micrograph of the *Z. officinale* sample (Figure 6a) reveals a relatively homogeneous microstructure composed of oval or polygonal structures with smooth surfaces embedded within a fibrous matrix. This morphology reflects the typical architecture of plant-derived materials, where bioactive phytoconstituents contribute to a porous, fibrous network. These structural characteristics are associated with good physicochemical stability and potential for effective interactions with other materials [53,54,55,56,57,58].

The SEM analysis of kaolinite (Figure 6b) shows a highly aggregated, layered microstructure composed of irregularly shaped, plate-like particles, representative of aluminosilicate minerals. The particle sizes range from 1 to 40 μm. The observed compact and flaky arrangement suggests pronounced interparticle associations contributing to the material’s inherent structural stability [19,79].

The SEM micrograph of the ZO–kaolinite system (Figure 6c) displays a heterogeneous microstructure with variable particle shapes and sizes ranging from 2 to 55 μm, with a notable concentration in the 5–30 μm range.

This morphology indicates robust interfacial interactions between the plant matrix (*Z. officinale*) and the inorganic component (kaolinite), where the former acts as a binding matrix and the latter provides additional stability through physical and chemical interactions, consistent with previously reported findings in the literature [79].

The integrated appearance of kaolinite platelets within the herbal matrix implies a structural incorporation rather than a simple physical mixture. These findings support the presence of interactions, such as hydrogen bonding, electrostatic forces, and potential partial intercalation of phytoconstituents into kaolinite interlayers [19,79]. The resultant new binary system presents a more compact and cohesive morphology.

The SEM analysis of the MZO carrier (Figure 6d) reveals a markedly different microstructure composed of uniformly dispersed spherical or near-spherical microcapsules, typically ranging from 1 to 10 μm. Numerous surface cavities, indicative of the spray drying process and rapid solvent evaporation, are visible. The overall morphology is consistent with maltodextrin-based encapsulation systems known to support phytoconstituent protection, structural homogeneity, and controlled release [54,80]. Compared to raw *Z. officinale* (Figure 6a), the MZO system demonstrates improved particle regularity and size distribution.

The SEM micrograph of the MZO–kaolinite system (Figure 6e) shows a compact and densely structured morphology, dominated by well-formed microcapsules with noticeably reduced porosity compared to the ZO–kaolinite system (Figure 6c). This transition indicates efficient encapsulation within the maltodextrin matrix. The fibrous texture characteristic of the herbal matrix is significantly diminished, and the flake-like features of kaolinite are less apparent. These features confirm the formation of a tricomponent composite system, likely stabilized through hydrogen bonding, electrostatic interactions, and matrix entrapment, which collectively enhance structural integrity and protect the encapsulated bioactive compounds.

In summary, the SEM observations illustrate the morphological progression from raw plant material to increasingly organized and encapsulated composite systems (MZO and MZO–kaolinite systems). The evolution in particle morphology and organization supports the successful preparation of the new multifunctional carrier systems (ZO–kaolinite and MZO–kaolinite systems). In the new tricomponent carrier system (MZO–kaolinite system), kaolinite contributes structural support, while maltodextrin enables effective encapsulation and protection of the bioactive components.

**Figure 7:**
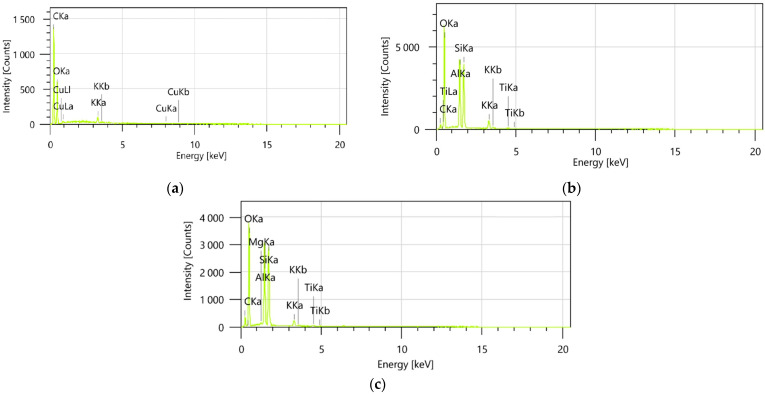
EDX analysis of the *Z. officinale* sample (**a**), kaolinite (**b**), and the ZO–kaolinite system (**c**). EDX: energy-dispersive X-ray.

EDX analysis provides a thorough examination of the elemental composition of the samples, offering valuable insights into their chemical characteristics and potential molecular interactions (Figure 7a–c).

EDX spectroscopy was utilized to determine the elemental distribution and confirm the successful preparation of the ZO–kaolinite system. The encapsulated samples (MZO and MZO–kaolinite systems) were not subjected to EDX analysis, as the high content of maltodextrin, composed predominantly of carbon and oxygen, would mask the detection of other characteristic elements and limit the interpretative value of the resulting spectra. Therefore, the binary system (ZO–kaolinite) was selected as the most representative for assessing the elemental-level interactions between the organic and inorganic components.

The EDX spectra for the ZO–kaolinite (Figure 7c) system revealed characteristic peaks corresponding to the elements present in both the *Z. officinale* sample (Figure 7a) and kaolinite (Figure 7b).

The presence of these distinct peaks verifies the coexistence of both components within the composite material. Furthermore, the observed spectral overlap between the peaks of the *Z. officinale* sample and kaolinite is indicative of their successful integration, suggesting strong intermolecular interactions at the atomic level. This overlap may also suggest the formation of new chemical bonds or interactions, such as hydrogen bonding, electrostatic forces, or van der Waals interactions, which could be responsible for stabilizing the ZO–kaolinite composite. These findings provide direct, empirical evidence of the effective incorporation of *Z. officinale* into the kaolinite matrix, reinforcing the conclusion that the ZO–kaolinite composite was successfully developed.

#### 3.5.4. DLS Analysis

**Figure 8:**
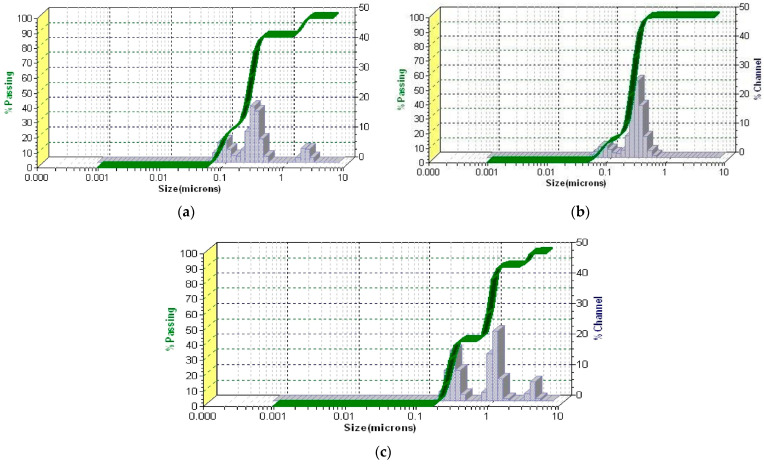
DLS pattern of the *Z. officinale* sample (**a**), kaolinite (**b**), and ZO–kaolinite system (**c**). DLS: dynamic light scattering.

The DLS analysis provides insights into the PSD of the ZO–kaolinite system and its raw materials (*Z. officinale* and kaolinite) in a dispersed medium. The results are presented in Figure 8a–c.

The DLS patterns of the *Z. officinale* sample (Figure 8a), kaolinite (Figure 8b), and the ZO–kaolinite system (Figure 8c) reveal distinct differences in the particle size distribution, polydispersity, and stability, reflecting their unique physicochemical characteristics. These differences are critical in determining the material’s behavior in colloidal systems and its potential applications.

The DLS pattern of the *Z. officinale* sample (Figure 8a) exhibits a broad particle size distribution, with the dominant peak centered in the 0.5–5 μm range. This is characteristic of plant-derived powders, which typically contain fibrous and irregularly shaped particles. The presence of larger aggregates (>5 μm) suggests agglomeration, likely driven by intermolecular interactions among starch granules, fibers, and secondary metabolites. Such aggregation can negatively impact suspension stability and limit the dispersibility of bioactive compounds. The polydispersity index (PDI) values further indicate a heterogeneous particle size distribution, with moderate polydispersity observed in medium-sized (PDI = 0.16) and smaller particles (PDI = 0.12), whereas larger particles exhibit a more monodisperse nature (PDI = 0.06). These findings suggest that *Z. officinale* alone has a non-uniform dispersion, which could compromise its performance in formulations requiring stable suspensions and consistent bioavailability.

In contrast, the DLS profile of kaolinite (Figure 8b) demonstrates a significantly narrower size distribution, indicative of improved uniformity and dispersion stability. The sharp peaks correspond to PDI values of 0.03 for larger particles, 0.099 for medium particles, and 0.11 for smaller particles, reflecting a highly monodisperse system with minimal aggregation. The well-defined particle size profile is attributed to kaolinite’s layered silicate structure and high surface area, which enhance its adsorption and dispersion properties. The uniformity in PSD makes kaolinite highly suitable for applications that require stable suspensions, controlled rheology, and efficient drug or bioactive compound delivery. The reduced aggregation observed in kaolinite, compared to the *Z. officinale* sample, suggests superior stabilization potential in composite systems.

The ZO–kaolinite system (Figure 8c) exhibits an intermediate behavior between *Z. officinale* and kaolinite, with a significant reduction in polydispersity and improved particle dispersion. The PDI values (0.03 for large particles, 0.099 for medium, and 0.11 for smaller particles) indicate a shift toward a more uniform particle size distribution, with reduced aggregation and enhanced stability compared to raw *Z. officinale*. This refinement in the size distribution suggests that kaolinite effectively stabilizes *Z. officinale* particles, likely through hydrogen bonding, electrostatic interactions, and adsorption of bioactive compounds onto the kaolinite surface. The incorporation of kaolinite into the ZO–kaolinite system offers several key advantages, primarily by enhancing particle stability, uniformity, and dispersion. The interaction between kaolinite and *Z. officinale* prevents excessive particle clustering, leading to a more stable suspension. This refined size distribution improves homogeneity, which is crucial for ensuring the controlled release and optimal bioavailability of active compounds. Additionally, the adsorption of bioactive components onto kaolinite surfaces reduces particle settling, promoting long-term stability. The narrower size distribution of medium and larger particles further suggests a more predictable and sustained release profile for phytochemicals, making the ZO–kaolinite system superior to *Z. officinale* alone. These findings highlight the ZO–kaolinite system as a well-balanced and stable composite, particularly suitable for applications requiring enhanced dispersion, bioavailability, and controlled release of bioactive compounds.

#### 3.5.5. PSD Analysis by Laser Diffraction

**Figure 9:**
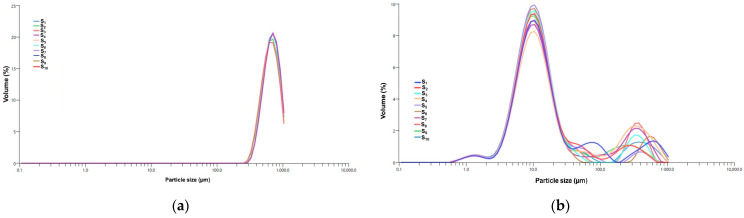
PSD curves from 10 consecutive measurements conducted over a two-minute period for the MZO carrier (**a**) and the MZO–kaolinite system (**b**). PSD: particle size distribution.

The PSD of the encapsulated samples, specifically the MZO carrier and MZO–kaolinite system, was assessed using laser diffraction analysis, as this technique is more suitable for characterizing particles larger than 10 μm, typically produced by spray drying. Because of the larger size range and dry powder form of these microparticles, laser diffraction provides more accurate and reproducible particle size measurements than DLS, which is limited to submicron suspensions. The resulting data, presented in Figure 9a,b, provide critical insights into both the PSD and the consistency of encapsulation across these samples.

The PSD analysis of the MZO carrier (Figure 9a) reveals a monomodal particle size distribution, as evidenced by the sharp, distinct peaks observed across all samples (S_1_ to S_10_). This consistency highlights a uniform particle population with minimal aggregation and variability across repeated measurements, confirming the reliability of the encapsulation process. The presence of a dominant peak within this narrow size range indicates the formation of microparticles, which are particularly advantageous for controlled-release applications. While larger particles may sometimes challenge dispersibility in aqueous media, the presence of maltodextrin as a stabilizing agent minimizes the excessive fragmentation of *Z. officinale* bioactive compounds, thereby extending their shelf life. These characteristics position the MZO carrier as a promising candidate for various applications, including powdered food formulations, nutraceuticals, and pharmaceuticals.

In contrast, the MZO–kaolinite system (Figure 9b) exhibits a bimodal particle size distribution, reflecting the presence of two distinct particle populations. The primary peak, occurring within the 1–10 μm range, corresponds to bioactive compounds encapsulated within the kaolinite structure [45,46,51,52,54]. The layered configuration and interlayer spacing of kaolinite facilitate the adsorption and intercalation of smaller *Z. officinale* constituents, forming well-dispersed microcapsules that contribute to enhanced stability and protection of bioactive compounds while maintaining a micro-sized distribution. Conversely, the secondary peak (~100–1000 μm) suggests partial aggregation or clustering, likely resulting from interactions between ginger phytoconstituents (polyphenols, essential oils, etc.) and the kaolinite surface. These interactions, facilitated by hydrogen bonding and electrostatic forces, may promote adhesion among kaolinite particles, leading to loosely bound aggregates rather than uniformly dispersed microcapsules [46,51,52,54,76,81]. Although this secondary peak accounts for less than 3% of the total volume, it still significantly influences the overall particle size metrics. Notably, the consistent overlap of the primary peak across samples (S_1_ to S_10_) underscores a highly reproducible encapsulation process, reinforcing the efficiency of kaolinite as a carrier for bioactive *Z. officinale* phytoconstituents.

Table 5 presents a comparative analysis of PSD metrics for the MZO and MZO–kaolinite systems, highlighting the influence of encapsulation on particle size.

The surface-weighted mean diameter (D [3,2]) for the MZO carrier is 771.21 μm, while the volume-weighted mean diameter (D [4,3]) measures 806.12 μm. In contrast, the MZO–kaolinite system exhibits significantly smaller values, with D [3,2] at 34.27 μm and D [4,3] at 64.45 μm. The cumulative distribution for the MZO carrier confirms a predominantly microparticle-based system, with d_10_ = 431.13 μm, d_50_ = 628.19 μm, and d_90_ = 879.23 μm. These values suggest a well-controlled encapsulation process, yielding particles primarily within the sub-1000 μm range, ideal for controlled-release applications [46,51,52,54,78,81].

In contrast, the MZO–kaolinite system demonstrates a broader particle size distribution, with d_10_ = 4.14 μm, d_50_ = 10.11 μm, and d_90_ = 90.41 μm. This wider range reflects the bimodal nature of the system, likely influenced by the complex structural interactions between the ZO–kaolinite composite and the maltodextrin matrix. Despite this variability, the smaller mean particle size in the MZO–kaolinite system suggests enhanced dispersion and improved stability, making it particularly suitable for applications requiring finely dispersed bioactive delivery [46,51,52,54,76,81]. Both encapsulated systems (MZO and MZO–kaolinite system) effectively reduce the mean diameter relative to the original *Z. officinale* particles, as confirmed by DLS analysis. Remarkably, an 18.5% reduction in the mean diameter of the MZO carrier and an even more significant 21.3% decrease for the ZO–kaolinite system were observed. These reductions suggest structural compaction and enhanced stabilization within the encapsulating matrix, leading to improved physicochemical stability and better retention of bioactive compounds. Furthermore, in the MZO–kaolinite system, the synergistic interaction between kaolinite and the biopolymeric matrix facilitates enhanced particle dispersion, reduces aggregation, and contributes to a more controlled and sustained release profile. These characteristics reinforce its potential as an advanced bioactive carrier system.

#### 3.5.6. Encapsulation Efficiency, Loading Capacity, and Encapsulation Yield

EE%, EC%, and EY% are critical parameters in assessing the quality, application potential, and economic feasibility of microencapsulation systems [53,54]. The encapsulation performance of the MZO carrier and the MZO–kaolinite system is summarized in Table 6, demonstrating their effectiveness in retaining bioactive compounds.

The EY% obtained in this study closely aligns with values reported in the literature, further validating the efficiency of the encapsulation process [46,51,54]. The MZO carrier exhibits an encapsulation yield of 63.61%, which falls within the expected range for maltodextrin-based encapsulation systems, as documented in previous studies on phytoconstituent encapsulation [46,51,54]. Similarly, the MZO–kaolinite system achieves a slightly lower EY% of 62.35%, which can be attributed to the synergistic interplay between kaolinite, phytoconstituents, and the biopolymeric matrix [54,58]. Encapsulation is a complex process influenced by multiple physicochemical interactions between the core bioactive compounds and the encapsulating matrix [46,47,51,54]. In the MZO carrier, encapsulation efficiency, stability, and release properties are governed by interactions between *Z. officinale* phytoconstituents and maltodextrin, while in the MZO–kaolinite system, these properties are further influenced by the presence of kaolinite, which introduces additional stabilization mechanisms. The MZO carrier relies on a combination of hydrogen bonding, hydrophobic interactions, van der Waals forces, and electrostatic interactions to encapsulate bioactive compounds effectively. Maltodextrin, rich in hydroxyl groups, establishes strong hydrogen bonds with hydroxyl-rich bioactive compounds, such as flavonoids, phenolic acids, and phenylpropanoids found in *Z. officinale*, enhancing the stability of encapsulation [46,51,52,54,76,81]. Additionally, non-polar bioactive compounds, such as fatty acids, hydrocarbons, sterols, and lipophilic phytochemicals, undergo hydrophobic interactions with the non-polar segments of maltodextrin, ensuring effective entrapment [46,51,52,54,76,81]. The van der Waals forces further reinforce the structural cohesion of the system, stabilizing phytoconstituents within the maltodextrin matrix [46,51,52,54,76,81]. Electrostatic interactions between polar bioactive molecules and maltodextrin’s charged regions contribute to the stability of microcapsules, preventing premature release [46,51,52,54,76,81]. Consequently, the MZO carrier forms a highly porous, spherical microstructure that facilitates enhanced solubility and controlled release. However, the increased porosity also heightens moisture absorption, which could impact long-term stability.

In contrast, the MZO–kaolinite system introduces kaolinite into the encapsulation process, significantly modifying the encapsulation mechanism. Kaolinite’s high surface energy fosters van der Waals interactions with maltodextrin, leading to the physical stabilization of encapsulated compounds [45,46,51,52,54,76,81]. Additionally, hydrogen bonding between the hydroxyl groups of maltodextrin and the hydroxyl-rich kaolinite surface reinforces the structural stability of the system [45,46,51,52,54,76,81]. Electrostatic attractions between kaolinite’s negatively charged surfaces and maltodextrin’s polar regions further enhance uniform dispersion, preventing aggregation and ensuring even distribution of encapsulated phytochemicals. Weak van der Waals forces help stabilize the matrix, preventing kaolinite aggregation while maintaining encapsulation efficiency [45,46,51,52,54,76,81]. The reduced porosity of the MZO–kaolinite system limits moisture absorption and enhances sustained release, making it particularly suitable for long-term storage and controlled delivery applications.

The encapsulation mechanisms of the MZO and MZO–kaolinite systems differ in terms of structural integrity, porosity, and stability due to the unique interactions between maltodextrin, *Z. officinale* biological compounds, and kaolinite. Furthermore, these findings, in conjunction with the results from SEM analysis, suggest that the MZO system is characterized by a highly porous, well-dispersed spherical microcapsule structure. This morphology enhances solubility and bioavailability but also increases moisture sensitivity. In contrast, the MZO–kaolinite system exhibits a denser, less porous structure, attributed to the additional hydrogen bonding and electrostatic interactions introduced by kaolinite. The high surface area and layered structure of kaolinite facilitate the physical adsorption and immobilization of phytochemicals, reducing their mobility while simultaneously improving thermal and environmental stability. Consequently, MZO microcapsules promote rapid release and enhanced solubility, whereas the MZO–kaolinite system provides sustained release, superior protection of bioactive compounds, and enhanced resistance to environmental factors. These characteristics make the MZO–kaolinite system more suitable for long-term applications requiring controlled release and improved stability.

### 3.6. Thermal Behavior

**Figure 10:**
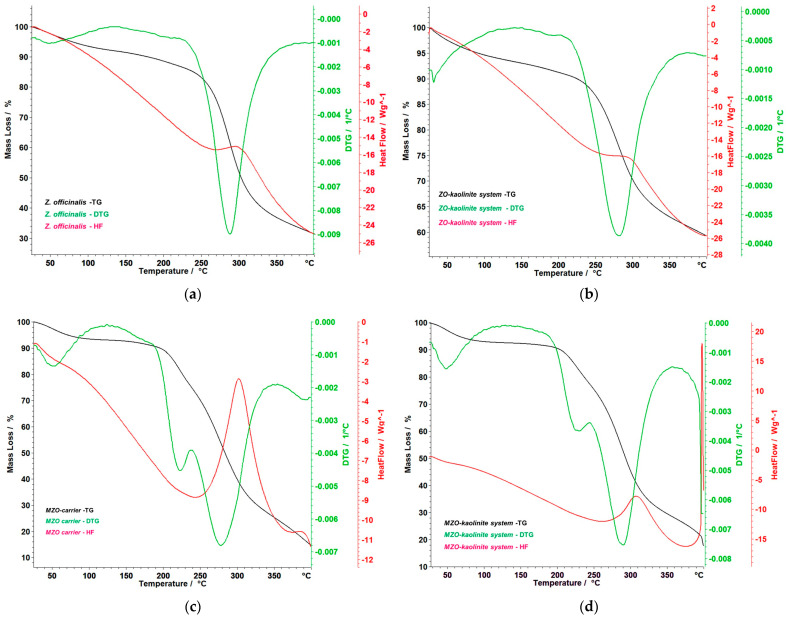
Thermoanalytical curves of the *Z. officinale* sample (**a**), ZO–kaolinite system (**b**), MZO carrier (**c**), and MZO–kaolinite system (**d**). DTG: derivative thermogravimetry; TG: thermogravimetry.

The thermal behavior of the *Z. officinale* sample and the ZO–kaolinite system were analyzed to assess their stability following encapsulation. This analysis is crucial for understanding the impact of encapsulation on the thermal properties of the bioactive components, as it provides insight into the thermal stability, decomposition temperatures, and potential changes in structural integrity that may occur during processing or under various environmental conditions. The results of the thermal analysis are presented in Figure 10a–d, which highlight the thermal profiles and stability characteristics of the samples, allowing for a comparative evaluation of the encapsulation effect on the overall thermal stability of the *Z. officinale* and ZO–kaolinite systems.

Figure 10a provides a detailed and insightful analysis of the thermogravimetry (TG), derivative thermogravimetry (DTG), and heat flow (HF) curves for the *Z. officinale* sample, covering a temperature range of 25–400 °C. These data are instrumental in understanding the thermal behavior of ginger, particularly its stability and the integrity of its bioactive compounds. The TG curve reveals a gradual mass loss of 4.31% around 100 °C, attributed to moisture evaporation. The most significant thermal event occurs between 226 °C and 343 °C, with a substantial mass loss of 48.25%. This degradation phase peaks at 288 °C, as indicated by the DTG curve, which highlights the decomposition of essential bioactive compounds, such as gingerols and shogaols. This breakdown is further corroborated by the HF curve, which exhibits a pronounced endothermic peak at 288 °C, indicating significant energy absorption during decomposition. Moreover, the recorded enthalpy change (ΔH) of 2181 mJ/g (or 2.181 J/g) reflects the considerable energy absorbed during the decomposition process. This finding emphasizes the intrinsic stability and resilience of ginger’s bioactive compounds when subjected to thermal stress. Overall, the total mass loss observed is a striking 67.81%, indicating the progressive degradation of ginger’s components at elevated temperatures, emphasizing the need for effective thermal management strategies to preserve its bioactivity.

Figure 10b presents the TG, DTG, and HF curves of the ZO–kaolinite system, illustrating its thermal stability and decomposition behavior within the 25–400 °C temperature range. The TG curve reveals an initial 4.59% mass loss between 38 and 100 °C, attributed to the evaporation of adsorbed moisture. This is a common feature of clay-based systems, where water molecules are loosely bound to the surface. A major thermal degradation phase occurs between 197 and 366 °C, where a 29.48% mass loss is observed, with a maximum degradation rate at 280 °C, as indicated by the DTG peak. This phase corresponds to the decomposition of organic bioactive compounds from *Z. officinale*, suggesting that the encapsulated ginger undergoes thermal degradation at a slightly higher temperature compared to the pure ginger sample. Notably, the HF curve of the ZO–kaolinite system exhibits a distinct exothermic peak at 298 °C, indicating that the degradation of the organic phase occurs as an exothermic reaction, likely due to oxidation of organic compounds in the presence of kaolinite. The total mass loss of 37.59% suggests that kaolinite provides a protective effect, reducing the overall thermal degradation compared to the Z. officinalis sample. This highlights kaolinite’s role as a stabilizing agent, making the *Z. officinale*–kaolinite system a promising carrier for thermally sensitive bioactive compounds.

Figure 10c presents the TG, DTG, and HF curves of the MZO carrier, demonstrating its thermal decomposition behavior within the 50–400 °C temperature range. The TG curve indicates an initial, gradual mass loss between 50 and 150 °C, which can be attributed to the evaporation of bound water and volatile compounds, indicating the moisture-retaining properties of the maltodextrin matrix. A major decomposition phase occurs between 183 and 344 °C, comprising two distinct but inseparable stages. In the first phase, (*i*) 183–238 °C, a mass loss of 15.96% is noted, coinciding with a DTG maximum at 220 °C. This phase marks the initial thermal degradation of maltodextrin and the early decomposition of bioactive compounds, underscoring the vulnerability of these components at lower heat exposure. In the second phase, (*ii*) 238–344 °C, a significant mass loss of 44.08% occurs, reaching a DTG peak at 282 °C. This stage represents the rapid breakdown of critical organic constituents, including gingerols, shogaols, and the maltodextrin matrix itself, indicating a complex interplay between the matrix and the encapsulated phytoconstituents. The HF curve reveals a notable endothermic event between 250 and 340 °C, accompanied by a ΔH of 6.637 J/g, indicating energy absorption during the decomposition process. This finding suggests that the encapsulated ginger components, as well as the maltodextrin, undergo structural disintegration within this temperature range. The overall mass loss of 63.90% suggests that while maltodextrin significantly contributes to thermal degradation, it also plays a crucial role in providing thermal stabilization. Notably, the degradation of ginger biomolecules occurs at higher temperatures compared to the *Z. officinale* sample, indicating that maltodextrin encapsulation imparts a protective effect. This enhancement not only delays the thermal degradation of *Z. officinale* biological compounds but also increases their stability, making maltodextrin an effective carrier in preserving the integrity and functionality of these essential compounds during processing and storage.

Figure 10d presents the thermoanalytical curves (TG, DTG, and HF) of the MZO–kaolinite system, illustrating its thermal stability and decomposition behavior in the 50–400 °C range. The TG curve indicates an initial mass loss due to the evaporation of water and volatile compounds, occurring between 38 and 72 °C, with a total mass loss of 4.90%. This phase represents the release of physically adsorbed moisture and lightweight volatile compounds, highlighting the MZO–kaolinite system’s ability to retain certain volatile components while expelling transient moisture. A significant decomposition process is observed between 192 and 339 °C, consisting of two overlapping stages. In the first stage, during the (*i*) 192–245 °C interval, there is a mass loss of 14.01%, marked by a DTG peak at 223 °C, suggesting the initial breakdown of organic compounds. In the second stage, (*ii*) 246–339 °C, a substantial mass loss of 44.85% is recorded, with a DTG peak at 290 °C, indicating a more rapid degradation of critical bioactive compounds, including gingerols, shogaols, and maltodextrin. Notably, the HF curve reveals a strong exothermic event starting at 397 °C, attributed to the structural transformation of kaolinite. However, the process is not entirely completed within the studied temperature range, indicating a gradual reaction extending beyond 400 °C. This aspect emphasizes the complex thermal dynamics at play within the system. The thermal stability profile suggests that kaolinite provides structural reinforcement, delaying the complete thermal degradation of ginger’s active compounds. Additionally, maltodextrin aids in controlled thermal release, helping to maintain the integrity of the phytochemicals. Together, these factors provide an added layer of stability that is highly beneficial for encapsulation applications, where the controlled degradation and release of ginger’s active components are desirable. These findings are valuable for optimizing processing, storage, and formulation strategies for encapsulated ginger-based functional ingredients, ensuring enhanced thermal protection and sustained bioactive release.

**Figure 11:**
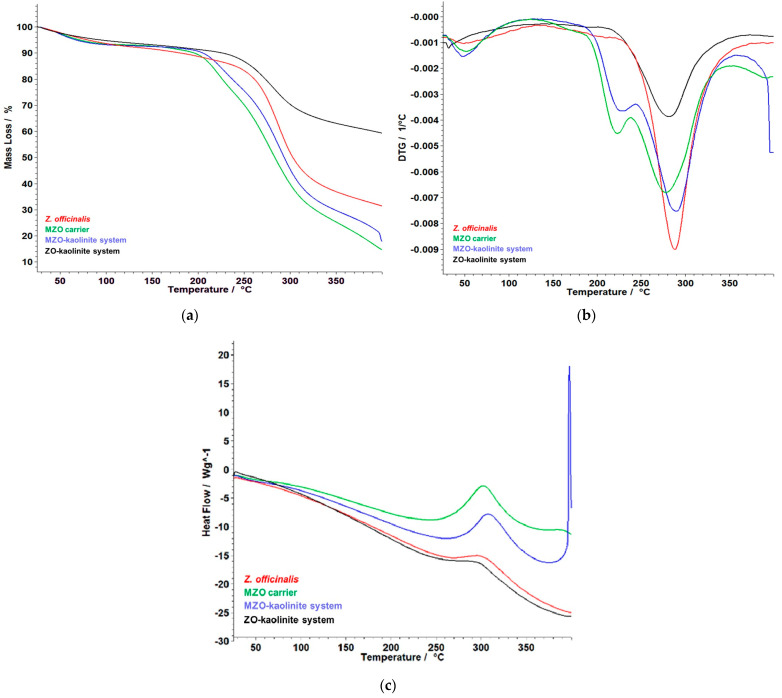
Comparative thermoanalytical curves for (**a**) TG, (**b**) DTG, and (**c**) HF of the *Z. officinale* sample, ZO–kaolinite system, MZO carrier, and MZO–kaolinite system samples. HF: heat flow.

A comparative evaluation of the thermal behaviors of all four samples reveals (Figure 11a–c) distinct differences in stability and degradation patterns.

The *Z. officinale* sample experiences the most rapid thermal degradation due to its high organic content, resulting in a total mass loss of 67.81%. In contrast, the MZO carrier demonstrates enhanced thermal stability, as maltodextrin acts as a protective matrix, delaying the decomposition of ginger’s bioactive compounds. The kaolinite-based systems (*Z. officinale*–kaolinite and MZO–kaolinite) exhibit the highest thermal resistance, attributed to kaolinite’s heat-resistant properties, which mitigate excessive mass loss. Among all formulations, the MZO–kaolinite system provides the most effective thermal protection, minimizing overall degradation while enabling a controlled release of bioactive compounds during thermal exposure. These findings emphasize the critical role of component selection in optimizing thermal stability, positioning encapsulation as a highly effective strategy for preserving ginger’s bioactivity in temperature-sensitive applications.

### 3.7. TPC and Estimation of Antioxidant Potential

**Figure 12:**
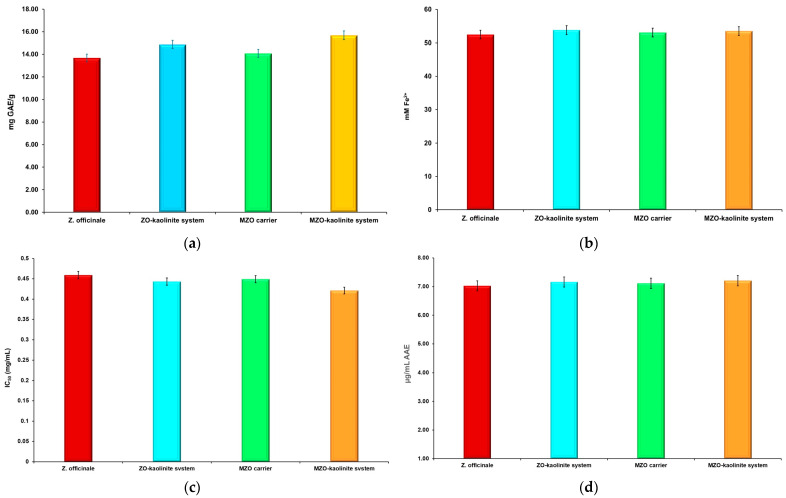
Results of the TPC (**a**), FRAP (**b**), DPPH (**c**), and TAC (**d**) assays for the *Z. officinale* sample, ZO–kaolinite system, MZO carrier, and MZO–kaolinite system. AAEs: ascorbic acid equivalents; DPPH: 2,2-Diphenyl-1-picrylhydrazyl; FRAP: ferric reducing antioxidant power; GAE: gallic acid equivalents; IC_50_; half-maximal inhibitory concentration; TAC: total antioxidant capacity; TPC: total phenolic content.

The antioxidant potential of the *Z. officinale* sample and the ZO–kaolinite system, both before and after encapsulation within the biopolymeric matrix, was comprehensively evaluated using four complementary assays: TPC, FRAP, DPPH radical scavenging activity (IC_50_), and TAC. This multifaceted approach offers a detailed insight into both the concentration of bioactive polyphenols and their functional redox activity, allowing for a thorough evaluation of their stability and efficacy before and after encapsulation in the maltodextrin matrix. Specifically, TPC quantifies the total polyphenol content, which is critical for antioxidant defense, while FRAP measures their electron-donating ability, reflecting their potential to alleviate oxidative stress. The DPPH assay further assesses radical scavenging efficiency by determining the IC_50_ values, and TAC offers an integrated view of the overall antioxidant activity. Collectively, these assays establish a robust framework for elucidating the impact of encapsulation on the antioxidant properties of these innovative carrier systems (MZO carrier and MZO–kaolinite system). The results are presented in Figure 12a–d.

The results detailed in Figure 12a–d significantly illustrate the robust antioxidant properties of *Z. officinale*. The analysis reveals a TPC of 13.676 ± 0.054 mg GAE/g, highlighting the rich reservoir of phenolic compounds known for their antioxidant efficacy. Additionally, the FRAP measurement of 52.461 ± 0.089 mM Fe^2+^ underscores the capacity of *Z. officinale* to effectively reduce ferric ions, thereby showcasing its potential as a powerful reducing agent. Moreover, the DPPH IC_50_ value of 0.459 ± 0.021 mg/mL indicates the extract’s notable scavenging ability. Finally, the TAC recorded at 7.023 ± 0.085 μg/mL AAE further substantiates the potent antioxidant capabilities of *Z. officinale*.

The incorporation of kaolinite to develop the ZO–kaolinite system significantly enhanced polyphenol retention and redox capacity, with TPC increasing to 14.867 ± 0.173 mg GAE/g (*p* < 0.05) and FRAP rising to 53.805 ± 0.066 mM Fe^2+^ (*p* < 0.05). The TAC for the ZO–kaolinite system improved to 7.126 ± 0.542 μg/mL AAE, further indicating enhanced antioxidant potential, while the DPPH IC_50_ slightly improved to 0.443 ± 0.031 mg/mL (*p* < 0.05).

According to the selected assays, the encapsulation of *Z. officinale* within a maltodextrin matrix in the newly developed MZO carrier resulted in moderate improvements in antioxidant activity compared with *Z. officinale*. Thus, the total phenolic content reached 14.072 ± 0.314 mg GAE/g. Although this increase was not statistically significant (*p* > 0.05), it supports the notion of a protective role played by the biopolymeric matrix in enhancing the retention of bioactive compounds, consistent with findings reported in the literature [46,51,52,54,76]. Additionally, the results of the FRAP assay indicated an increase to 53.091 ± 0.019 mM Fe^2+^, although the changes in DPPH IC_50_ (0.449 ± 0.116 mg/mL) and TAC (7.215 ± 0.037 μg/mL AAE) were not statistically significant (*p* > 0.05). This aligns with the existing literature that highlights the ability of biopolymers to encapsulate and protect sensitive phytochemicals from degradation, thus possibly improving their bioavailability [46,51,52,54,76]. Although the differences observed were not statistically significant (*p* > 0.05), the trends in antioxidant activity suggest that the MZO carrier may still provide a slight advantage. The lack of statistical significance could be attributed to the experimental procedure used in this study and or the specific assays used. However, these results suggest that the MZO carrier retains a comparable ability to scavenge free radicals, which is crucial for assessing the potential health benefits of such formulations. Furthermore, the interaction of compounds within the MZO carrier may lead to complex behavior in terms of antioxidant activity. The biopolymer may not only protect but also interact with the antioxidant compounds from this sample in a way that influences their activity. This complexity could mask significant differences in some assays while still reflecting an overall increase in antioxidant potential. Nonetheless, the results support the hypothesis that the biopolymeric matrix could play a beneficial role in preserving bioactive compounds, thereby enhancing their functional properties [46,51,52,54,76].

Most notably, the MZO–kaolinite system demonstrated the highest overall antioxidant performance, with TPC of 15.678 ± 0.019 mg GAE/g (*p* < 0.01), FRAP of 54.487 ± 0.023 mM Fe^2+^ (*p* < 0.01), the lowest DPPH IC_50_ of 0.42 ± 0.072 mg/mL (*p* < 0.01), and TAC of 7.508 ± 0.063 μg/mL AAE (*p* < 0.05). These statistically significant improvements confirm that the synergistic combination of maltodextrin and kaolinite not only enhances polyphenol retention but also boosts electron-donating capacity and free radical scavenging efficiency.

### 3.8. Antimicrobial Screening

The antibacterial properties of *Z. officinale* and the newly developed ZO–kaolinite system were systematically evaluated following their encapsulation within a maltodextrin matrix. The antimicrobial efficacy was assessed by measuring inhibition zone (IZ) diameters and comparing them to kaolinite alone, as well as positive (Gentamicin) and negative (DMSO) controls. Antimicrobial activity was tested against a diverse range of Gram-positive and Gram-negative pathogenic bacteria, including *S. aureus*, *E. faecalis*, *B. cereus*, *K. pneumoniae*, *P. aeruginosa*, and *E. coli*, using the agar well diffusion method.

The antimicrobial performance of each sample was evaluated at concentrations of 100–200 μg/mL, and the results demonstrated significant differences in efficacy. The data are presented in Table 7.

The results demonstrated that the MZO–kaolinite system exhibited the highest antimicrobial activity, with IZs ranging from 48.06 ± 0.27 mm (*B. cereus*) to 82.03 ± 0.32 mm (*K. pneumoniae*) at 200 μg/mL, significantly surpassing all the other tested samples (*p* < 0.05). Notably, its IZ values consistently exceeded those of Gentamicin, highlighting its superior antibacterial efficacy. Against Gram-positive bacteria, the MZO–kaolinite system (77.26 ± 0.58 mm) exhibited an IZ approximately three times larger than that of Gentamicin (22.18 ± 0.22 mm) against *S. aureus*, while the ZO–kaolinite system (73.33 ± 0.41 mm) also demonstrated significant activity. A similar trend was observed against *E. faecalis*, where the MZO–kaolinite system (52.47 ± 0.43 mm) significantly outperformed Gentamicin (20.64 ± 0.17 mm), followed by the ZO–kaolinite system (40.13 ± 0.32 mm), MZO carrier (27.05 ± 0.34 mm), and *Z. officinale* extract (23.73 ± 0.08 mm). Against *B. cereus*, the MZO–kaolinite system (48.06 ± 0.27 mm) and ZO–kaolinite system (43.77 ± 0.33 mm) showed the highest efficacy, while kaolinite exhibited the lowest inhibition (18.22 ± 0.16 mm), though still slightly superior to Gentamicin (17.03 ± 0.24 mm).

For Gram-negative bacteria, the MZO–kaolinite system maintained its superior efficacy, exhibiting the highest inhibition against *P. aeruginosa* (74.95 ± 0.14 mm), *K. pneumoniae* (82.03 ± 0.32 mm), and *E. coli* (50.63 ± 0.52 mm). Against *P. aeruginosa*, the MZO–kaolinite system significantly outperformed the ZO–kaolinite system (69.02 ± 0.38 mm), the MZO carrier (55.02 ± 0.41 mm), and the *Z. officinale* extract (53.47 ± 0.32 mm), while kaolinite (35.62 ± 0.51 mm) exhibited greater inhibition than Gentamicin (30.54 ± 0.21 mm). Against *K. pneumoniae*, the MZO–kaolinite system (82.03 ± 0.32 mm) recorded the highest inhibition, surpassing the ZO–kaolinite system (78.72 ± 0.42 mm), the MZO carrier (68.75 ± 0.26 mm), and the *Z. officinale* extract (66.47 ± 0.18 mm). Kaolinite (21.78 ± 0.33 mm) exhibited the lowest inhibition but remained more effective than Gentamicin (15.75 ± 0.23 mm). A similar pattern was observed against *E. coli*, where the MZO–kaolinite system (50.63 ± 0.52 mm) exhibited the highest inhibition, followed by the ZO–kaolinite system (43.02 ± 0.23 mm), the MZO carrier (23.85 ± 0.16 mm), and the *Z. officinale* extract (21.12 ± 0.07 mm). Interestingly, kaolinite (31.22 ± 0.18 mm) exhibited greater antimicrobial activity than *Z. officinale* and the MZO carrier, even surpassing Gentamicin (20.59 ± 0.32 mm). Notably, at lower concentrations (75–100 μg/mL), the antimicrobial activity of the *Z. officinale* extract, kaolinite, and the MZO carrier was lower than that of Gentamicin against *E. coli*. However, the ZO–kaolinite system exhibited an IZ of 21.26 ± 0.31 mm at 75 μg/mL, comparable to Gentamicin, and outperformed it at higher concentrations. The MZO–kaolinite system consistently demonstrated IZs larger than those of Gentamicin across all tested concentrations against this bacterial strain, confirming its superior and concentration-dependent antimicrobial efficacy.

To further validate the antibacterial efficacy of all the samples, including *Z. officinale*, kaolinite, the newly developed ZO–kaolinite system, the MZO carrier, and the MZO–kaolinite system, MICs and MBCs were determined against all bacterial strains tested. This analysis provides critical insights into the effectiveness of each sample in inhibiting bacterial growth and elucidates their potential as antimicrobial agents in the context of the strains utilized in this study. The results obtained are displayed in Table 8.

The MIC and MBC assays further validated these findings. The MZO–kaolinite system consistently exhibited the lowest MIC and MBC values across all strains (MIC: 0.26 ± 0.09 to 1.34 ± 0.12 μg/mL), indicating superior antimicrobial potency. Statistical analysis (*p* < 0.05) confirmed its significantly enhanced efficacy over the *Z. officinale* extract, kaolinite, the MZO carrier, and the ZO–kaolinite system. Notably, the MIC and MBC values correlated with the IZ data, reinforcing the system’s broad-spectrum antimicrobial potential.

### 3.9. Cell Viability Assay

**Figure 13:**
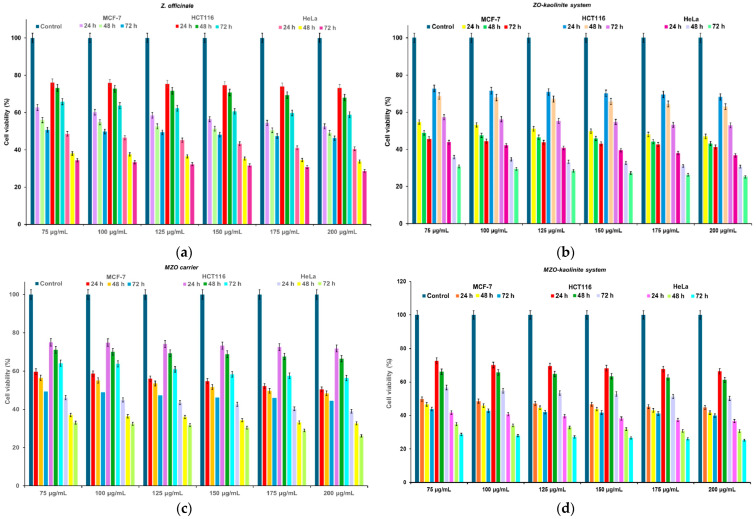
Viability of MCF-7, HCT-116, and HeLa cells, assessed at 24, 48, and 72 h after co-incubation with varying concentrations of the *Z. officinale* sample (**a**), the ZO–kaolinite system (**b**), the MZO carrier (**c**), and the MZO–kaolinite system (**d**). Positive control wells included untreated cells, MTT solution, and DMSO. Data are presented as mean ± SD (*n* = 3).

Figure 13a–d present the results of cell viability assessments derived from the MTT assays conducted on three cancer cell lines: MCF-7 (breast cancer), HCT-116 (colorectal cancer), and HeLa (cervical cancer).

This study evaluates the cytotoxic effects of the *Z. officinale* sample (Figure 13a), the ZO–kaolinite system (Figure 13b), the MZO carrier (Figure 13c), and the MZO–kaolinite system (Figure 13d) across a concentration range of 75–200 μg/mL. Cell viability percentages serve as indicators of metabolic activity, where higher viability reflects reduced cytotoxicity and increased mitochondrial function, while lower viability signifies greater cytotoxic effects.

Cytotoxicity analysis confirms the anticancer potential of the *Z. officinale* extracts, and their newly developed carrier systems provide strong evidence of their anticancer potential.

Across all treatment conditions, a dose- and time-dependent decline in cell viability was observed (*p* < 0.01), highlighting the critical influence of concentration and exposure duration on therapeutic efficacy. Notably, the ZO–kaolinite and MZO–kaolinite systems exhibited significantly enhanced cytotoxicity compared to *Z. officinale* alone (*p* < 0.01), underscoring the role of kaolinite as an effective carrier in facilitating bioactive compound delivery. In the ZO–kaolinite system, kaolinite likely enhances compound stability and cellular uptake through adsorption and controlled release mechanisms. Meanwhile, in the MZO–kaolinite system, the additional encapsulation within a maltodextrin matrix via spray drying further improves particle dispersion, bioavailability, and sustained release, thereby amplifying therapeutic efficacy.

**Figure 14:**
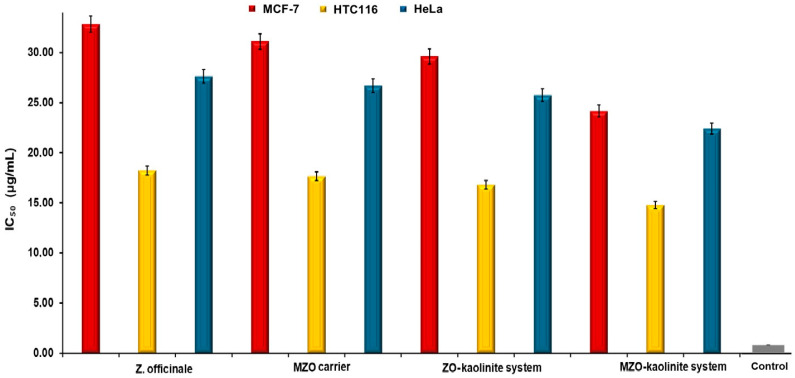
In vitro cytotoxicity of the *Z. officinale* sample, the ZO–kaolinite system, the MZO carrier, and the MZO–kaolinite system, as a function of concentration against MCF-7, HCT-116, and HeLa cells (after 24 h). Data are represented as mean ± SD (*n* = 3).

The cytotoxic effects of different formulations were evaluated across three cancer cell lines (MCF-7, HCT-116, and HeLa), based on their IC_50_ values. The results are presented in Figure 14.

The MZO–kaolinite system exhibited the lowest IC_50_ values across all tested cell lines, indicating superior cytotoxic activity compared to the other samples (*Z. officinale*, ZO–kaolinite system, and MZO, respectively). In MCF-7 cells, the IC_50_ values were 24.15 ± 0.032 μg/mL (MZO–kaolinite), 29.61 ± 0.053 μg/mL (ZO–kaolinite), 31.11 ± 0.018 μg/mL (MZO carrier), and 32.84 ± 0.092 μg/mL (*Z. officinale*). HCT-116 cells demonstrated the highest sensitivity, with IC_50_ values of 14.78 ± 0.107 μg/mL (MZO–kaolinite), 16.81 ± 0.137 μg/mL (ZO–kaolinite), 17.06 ± 0.152 μg/mL (MZO carrier), and 18.21 ± 0.117 μg/mL (*Z. officinale*). Similarly, in HeLa cells, the IC_50_ values followed the same trend: 22.42 ± 0.052 μg/mL (MZO–kaolinite), 25.77 ± 0.026 μg/mL (ZO–kaolinite), 26.71 ± 0.049 μg/mL (MZO carrier), and 27.64 ± 0.165 μg/mL (*Z. officinale*). These results confirm a clear trend in cytotoxic efficacy, with HCT-116 cells being the most sensitive and MCF-7 cells the least responsive. Among all the tested samples, the MZO–kaolinite system exhibited the greatest cytotoxicity, as evidenced by its significantly lower IC_50_ values across all three cell lines (*p* < 0.01). Additionally, its cytotoxic effects were significantly greater than those of the MZO carrier and the ZO–kaolinite system (*p* < 0.05), suggesting that the synergistic combination of maltodextrin and kaolinite enhances bioactivity. This improvement is likely to result from increased bioavailability, enhanced intracellular retention, and a controlled-release profile. Furthermore, the ZO–kaolinite and MZO–kaolinite systems exhibited significantly lower IC_50_ values than *Z. officinale* alone (*p* < 0.01), highlighting the enhanced cytotoxic potential of these newly prepared engineered carrier systems.

## 4. Discussion

### 4.1. Phytochemical Screening

*Terpenoids*, constituting 17.14% of the total phytoconstituents in *Z. officinale*, represent the predominant class of bioactive compounds identified (Figure 2; Table 2). Renowned for their extensive therapeutic potential, terpenoids exhibit a wide range of pharmacological properties, including antitumor, antimicrobial, antiviral, analgesic, antispasmodic, anti-inflammatory, cardioprotective, antihyperglycemic, and immunomodulatory effects [85]. Their diverse bioactivities highlight their significant role in health promotion and disease prevention, positioning them as promising candidates for further research and potential therapeutic applications [85].

*Amino acids* constitute the second most abundant class of metabolites in *Z. officinale*, accounting for 16.19% of the plant’s total phytochemical profile (Figure 2; Table 2). A total of 14 amino acids were identified in the ginger extract, with essential amino acids, including valine, leucine, threonine, methionine, isoleucine, histidine, phenylalanine, tryptophan, and arginine, comprising 52.94% of the total. The remaining 47.06% consists of non-essential amino acids, such as glycine, alanine, aspartic acid, serine, proline, glutamate, and tyrosine [86,87,88,89]. This balanced amino acid composition is particularly noteworthy, as these biomolecules have been widely recognized for their antitumoral, antiproliferative, and immunomodulatory properties [86,87,88,89], reinforcing their significance in potential therapeutic applications.

*Flavonoids*, comprising 4.76% of the plant’s phytochemicals, are potent bioactive metabolites renowned for their extensive range of biological activities (Figure 2; Table 2). These include significant antioxidant, antiviral, antimicrobial, antitumor, cardioprotective, and neuroprotective effects. The diverse therapeutic potential of flavonoids highlights their critical role in health promotion and disease mitigation, making them a valuable target for further investigation and potential therapeutic development [90].

*Fatty acids* constitute a significant 13.34% of the total phytochemicals identified in the *Z. officinale* sample, underscoring their pivotal role within the plant’s bioactive profile (Figure 2; Table 2). This group includes 11 saturated fatty acids (palmitic acid, behenic acid, lignoceric acid, undecanoic acid, lauric acid, myristic acid, caprylic acid, stearic acid, margaric acid, arachidic acid, and capric acid) and three monounsaturated fatty acids, comprising one essential ω-6 fatty acid (linoleic acid) and two non-essential fatty acids: ω-7 (palmitoleic acid) and ω-9 (oleic acid) (Table 2). These fatty acids are renowned for their diverse and multifunctional therapeutic properties, including potent antioxidant, antimicrobial, anti-inflammatory, neuroprotective, and cardioprotective effects [91].

*Phytosterols* account for 2.85% of the total phytoconstituents identified, showcasing their remarkable therapeutic potential (Figure 2; Table 2). These bioactive compounds are known to exhibit a wide array of beneficial effects, including antioxidant, neuroprotective, cardioprotective, anti-inflammatory, antitumor, and immunomodulatory activities [92].

*Phenolic acids* are renowned for their diverse and potent therapeutic properties, including antioxidant, antibacterial, antitumor, anti-inflammatory, anti-allergic, antidiabetic, cardioprotective, and neuroprotective effects [93,94]. These bioactive compounds play a pivotal role in promoting health and preventing disease, further underscoring their importance in both pharmaceutical and nutraceutical applications [95,96].

*Diarylheptanoids* account for 3.80% of the total phytochemicals identified in the ginger samples, underscoring their critical role in the plant’s bioactive composition (Figure 2; Table 2). These compounds exhibit an impressive spectrum of therapeutic properties, including anti-inflammatory, antitumor, antioxidant, anti-estrogen, hepatoprotective, anti-leishmanial, and neuroprotective effects [95,96,97,98,99,100]. Among them, gingerenone A stands out for its reported ability to induce antiproliferation and senescence in breast cancer cells, emphasizing its promising potential in oncology [96]. Similarly, hexahydrocurcumin has demonstrated equal or superior bioactivity compared to curcumin, in both in vitro and in vivo studies, offering multifaceted benefits, such as antioxidant, anti-inflammatory, antitumor, and cardiovascular protective properties [97,98]. Additionally, diacetoxy-6-gingerdiol exhibits robust anti-inflammatory and antioxidant activities, while dihydroxycurcumin has shown potent antibacterial, antimicrobial, and antibiofilm effects [99].

*Gingerols*, *shogaols*, and *paradols* constitute 15.23% of the phytochemicals identified in ginger and are key contributors to its medicinal value (Figure 2; Table 2). Gingerol and its related compounds exhibit potent antioxidant, antitumor, anti-inflammatory, analgesic, antimicrobial, and hepatoprotective activities, making them highly versatile bioactive agents [7,8,9,10,14,23,24,25,26,28,68,69,101]. Similarly, paradols are renowned for their antioxidant, anticancer, and antimicrobial properties [7,8,9,10,14,23,24,25,26,28,68,69,101]. Shogaols, particularly [6]-shogaol, stand out for their robust anticancer effects, including inhibition of cell invasion, reduction in matrix metalloproteinase-9 (MMP-9) expression, and antiproliferative activities, along with strong antioxidant and anti-inflammatory effects [102,103]. Collectively, these compounds underscore ginger’s potential as a therapeutic agent for addressing oxidative stress, inflammation, cancer, microbial infections, and liver protection.

The *phenylpropanoid* eugenyl acetate displays antioxidant, antimicrobial, anti-inflammatory, analgesic, cytotoxic, insecticidal, and neuroprotective effects [104].

### 4.2. Antioxidant Potential

Our findings reinforce *Z. officinale* as a potent source of natural antioxidants, particularly rich in polyphenolic constituents with demonstrated free radical scavenging capacity [7,8,10,13,23,26,27,28,68,69,83,101,102]. To enhance the stability and bioactivity of these phytoconstituents, we developed and comparatively evaluated three carrier systems with increasing structural complexity: ZO–kaolinite, MZO, and MZO–kaolinite. This comparative analysis revealed distinct differences in antioxidant performance, culminating in a synergistic enhancement in the most complex system.

In the ZO–kaolinite system, kaolinite, a naturally occurring clay with a layered aluminosilicate structure, high thermal stability, and large specific surface area, acted as both an adsorbent and intercalation medium for polyphenolic compounds. These interactions protected the bioactive phytoconstituents from oxidative degradation and significantly enhanced the antioxidant potential of *Z. officinale* [58,105]. This system demonstrated greater radical scavenging activity than the MZO system, underscoring kaolinite’s stabilizing capacity. By contrast, the MZO system, in which *Z. officinale* was encapsulated solely in a maltodextrin matrix, provided improved solubility and moderate stabilization. Although this system exhibited enhanced antioxidant capacity compared to *Z. officinale*, its performance was lower than that of the new ZO–kaolinite system.

The most pronounced antioxidant activity was observed in the MZO–kaolinite system, which resulted from encapsulating the ZO–kaolinite complex within a maltodextrin matrix. This ternary system leveraged the synergistic interaction between kaolinite and maltodextrin, combining kaolinite’s structural stabilization with maltodextrin’s matrix-forming and solubility-enhancing properties. Encapsulation in maltodextrin further protected the kaolinite-bound phytoconstituents and facilitated improved dispersion and controlled release of bioactive compounds.

Our results reveal a strong positive correlation between TPC and electron-donating capacity, particularly pronounced in the MZO–kaolinite system. This correlation supports the hypothesis that the system’s superior antioxidant performance arises not merely from additive effects but from a synergistic interaction that enhances phenolic stability, bioavailability, and radical scavenging efficiency [46,51,52,54,75,76,81]. Moreover, antioxidant assays (DPPH, ABTS, FRAP) confirmed that the MZO–kaolinite system exhibited the highest phenolic retention and the most potent antioxidant activity among the three systems. This outcome underscores the benefit of integrating both inorganic (kaolinite) and organic (maltodextrin) carriers into a hierarchical delivery platform. The maltodextrin matrix surrounding the ZO–kaolinite complex likely minimizes oxidative degradation and promotes a more uniform release of phytoconstituents in reactive environments.

Taken together, these findings demonstrate that while the MZO and ZO–kaolinite systems individually offer protective and functional benefits, their integration into the MZO–kaolinite system results in a multifunctional delivery vehicle with markedly enhanced antioxidant performance. This ternary formulation holds significant promise for applications in nutraceutical and biomedical fields aimed at mitigating oxidative stress and improving the stability and delivery of bioactive compounds.

### 4.3. Antimicrobial Screening

The selection of specific bacterial strains (*S. aureus*, *E. faecalis*, *B. cereus*, *P. aeruginosa*, *K. pneumoniae*, and *E. coli*) is scientifically justified based on their clinical relevance, virulence potential, and increasing antibiotic resistance profiles [16,17]. These strains represent both Gram-positive and Gram-negative bacteria that are frequently implicated in nosocomial infections, foodborne illnesses, and community-acquired diseases. *S. aureus*, particularly Methicillin-resistant strains (MRSA), is a major etiological agent in skin, respiratory tract, and bloodstream infections. *E. faecalis* is commonly associated with urinary tract infections (UTIs), endocarditis, and intra-abdominal infections, with many strains exhibiting Vancomycin resistance (VRE) [16,17]. *B. cereus* is a spore-forming bacterium linked to foodborne gastroenteritis and opportunistic infections, particularly in immunocompromised individuals [16,17]. Among Gram-negative bacteria, *P. aeruginosa* is a notorious multidrug-resistant opportunistic pathogen responsible for severe infections in burn patients and immunocompromised hosts. *K. pneumoniae* is a leading cause of hospital-acquired pneumonia, UTIs, and sepsis, with many strains developing resistance to carbapenems (CRKP) [16,17]. *E. coli* includes pathogenic variants such as enterotoxigenic (ETEC), enterohemorrhagic (EHEC), and uropathogenic (UPEC) strains, which are involved in gastrointestinal diseases, UTIs, and systemic infections [16,17]. These strains were selected to provide a comprehensive assessment of antimicrobial efficacy against clinically significant pathogens with diverse resistance mechanisms, including biofilm formation, efflux pump activity, and enzymatic degradation of antibiotics [16,17].

To evaluate the antimicrobial potential of our novel *Z. officinale*-based formulations, three systems were comparatively analyzed: MZO, ZO–kaolinite, and MZO–kaolinite.

The results demonstrated that MZO–kaolinite exhibited the most pronounced and broad-spectrum antibacterial activity, outperforming both MZO and ZO–kaolinite in terms of IZ diameters and MICs. This enhanced efficacy is attributed to tripartite synergism among kaolinite, ginger-derived bioactive compounds (notably polyphenols, terpenoids, and other phytoconstituents) [10,11,12,13,14,80,106,107,108], and the maltodextrin matrix. Kaolinite, a layered silicate mineral, functions as both an adsorbent and a stabilizing carrier, facilitating prolonged release and enhancing surface-mediated interactions with bacterial cells [37,38,39,40,41,42,43,44,45,58,79,105,109,110,111]. Maltodextrin, as a biopolymeric encapsulant, protects bioactive compounds from oxidative degradation, enhances solubility, and modulates release kinetics to sustain antimicrobial activity [46,54,75].

In contrast, the ZO–kaolinite system, while more effective than the *Z. officinale* extract alone, exhibited lower efficacy compared to MZO–kaolinite. This is likely due to the absence of maltodextrin’s protective and controlled-release functions. The MZO formulation also demonstrated substantial antimicrobial activity, primarily owing to the preserved bioactivity of encapsulated ginger constituents. However, its performance was comparatively reduced relative to MZO–kaolinite, potentially due to the lack of surface stabilization and interaction enhancement provided by kaolinite.

Mechanistically, the MZO–kaolinite system likely exerts antimicrobial effects through multiple pathways, including membrane disruption, inhibition of biofilm formation, and interference with microbial metabolic processes, consistent with the known antibacterial mechanisms of *Z. officinale* [10,11,12,13,14,80,106,107,108]. These multimodal actions likely contribute to its superior efficacy against resistant bacterial strains.

In summary, our findings demonstrate that sequential functionalization, first with kaolinite, followed by encapsulation in a maltodextrin matrix, markedly enhances the antimicrobial efficacy of *Z. officinale* phytochemicals. The resulting MZO–kaolinite system emerges as a potent, broad-spectrum antimicrobial platform with promising applications in food safety and biomedical contexts, particularly where resistance to conventional antibiotics poses a significant threat.

### 4.4. Cytotoxic Activity

*Z. officinale* exhibits well-documented intrinsic cytotoxic properties, primarily attributed to its diverse array of bioactive constituents, including gingerols, shogaols, paradols, and terpenes. These phytochemicals exert anticancer effects through multiple converging mechanisms, notably the induction of apoptosis, generation of intracellular oxidative stress, and arrest of the cell cycle [7,14,23,24,25,26,96,103,112,113,114,115,116]. Several studies have demonstrated that *Z. officinale* treatment significantly increases mitochondrial reactive oxygen species (ROS) levels, activates executioner caspases (e.g., caspase-3 and caspase-9), and promotes deoxyribonucleic acid (DNA) fragmentation, collectively contributing to programmed cell death [7,14,23,24,25,26,96,103,112,113,114,115,116]. Moreover, *Z. officinale* downregulates pro-inflammatory cytokines, such as interleukin-6 (IL-6) and tumor necrosis factor-alpha (TNF-α), by inhibiting the NF-κB signaling pathway, thereby attenuating inflammation-associated tumorigenesis [7,14,23,24,25,26,96,103,112,113,114,115,116].

To enhance its bioactivity and address inherent delivery limitations, *Z. officinale* was incorporated into three distinct delivery systems: MZO, ZO–kaolinite, and MZO–kaolinite. The cytotoxic efficacy of these formulations was evaluated in three cancer cell lines: MCF-7, HCT-116, and HeLa.

The ZO–kaolinite system demonstrated significantly enhanced cytotoxic activity compared to native *Z. officinale*. Viability assays showed markedly reduced cell survival across all concentrations and time points (*p* < 0.01), highlighting kaolinite’s role in improving cellular uptake and intracellular retention of phytoconstituents [41,43,44,45,58,79,117,118,119,120,121,122,123,124]. At 200 μg/mL, after 72 h, cell viability decreased to 41.27% in MCF-7, 52.88% in HCT-116, and 25.27% in HeLa cells, suggesting heightened sensitivity in colorectal and cervical cancer cells. These results suggest a correlation between kaolinite-mediated delivery and increased susceptibility of highly proliferative, metabolically active cell types [118,119,120,121].

In contrast, MZO exhibited only moderate cytotoxicity. While maltodextrin effectively stabilized and solubilized the *Z. officinale* phytoconstituents, its role was primarily passive [54,75]. MZO-treated cells showed significantly higher viability than those treated with ZO–kaolinite (*p* < 0.05). These findings support the conclusion that maltodextrin alone does not significantly enhance cellular uptake or control intracellular release [54,75,125,126,127]. Among the tested cell lines, MCF-7 cells displayed the highest survival rates, reflecting their comparatively lower sensitivity to oxidative stress.

The MZO–kaolinite system exhibited the most potent cytotoxic activity among all the tested formulations. This ternary system synergistically combined the stabilizing properties of maltodextrin with the adsorptive and delivery-enhancing characteristics of kaolinite [54,75,117,118,119,120,121,125,126,127]. After 72 h, at 200 μg/mL, cell viability was reduced to 40.12% (MCF-7), 50.25% (HCT-116), and 25.27% (HeLa), with statistically significant improvements over both the MZO and ZO–kaolinite formulations (*p* < 0.001). These findings suggest a synergistic interaction wherein kaolinite facilitates cellular uptake and intracellular retention, while maltodextrin contributes to structural stability and sustained release [54,75,117,118,119,120,121,125,126,127].

The enhanced cytotoxicity observed in the kaolinite-containing systems (ZO–kaolinite and MZO–kaolinite) likely results from a combination of improved intracellular accumulation, sustained phytoconstituent release, and increased ROS-mediated mitochondrial disruption [41,43,44,45,58,79,117,118,119,120,121,122]. A time-dependent decline in cell viability and dose-response regression analyses (*R*^2^ > 0.95 for all systems) confirmed robust and dose-proportional cytotoxic effects across all formulations. Notably, the strongest effects were consistently observed in HCT-116 and HeLa cells, suggesting that cell line-specific redox balance and metabolic profiles play critical roles in responsiveness to phytochemical-induced apoptosis. Statistical comparisons confirmed significantly lower viability in HCT-116 and HeLa cells, compared to MCF-7, across all formulations (*p* < 0.05), consistent with their higher sensitivity to ROS-mediated apoptosis.

Comparative analysis of cytotoxic activity among the tested delivery systems revealed distinct performance profiles. *Z. officinale* alone exerted baseline cytotoxicity through apoptosis and oxidative stress pathways. Encapsulation in maltodextrin (MZO) improved solubility and stability but provided only a moderate enhancement in cytotoxic efficacy, indicating that maltodextrin primarily preserves rather than amplifies bioactivity. The ZO–kaolinite system exhibited significantly greater cytotoxic potential due to kaolinite’s ability to enhance cellular delivery and retention. The MZO–kaolinite system emerged as the most effective, combining maltodextrin’s stabilizing effects with kaolinite’s delivery-enhancing capabilities. This dual-functional carrier facilitated controlled release, improved bioavailability, and prolonged intracellular exposure to cytotoxic agents, resulting in the greatest reduction in cancer cell viability. These findings underscore the superior therapeutic efficacy of the MZO–kaolinite system and its promise as a platform for anticancer delivery of *Z. officinale* phytoconstituents.

The superior performance of the MZO–kaolinite system highlights the synergistic interplay between maltodextrin’s stabilizing matrix and kaolinite’s intracellular delivery-enhancing properties. The significantly lower IC_50_ values observed for the MZO–kaolinite system (*p* < 0.01) further confirm its potential as an optimized platform for phytochemical-based anticancer therapies.

However, although kaolinite is generally recognized as safe (GRAS), its long-term biocompatibility, potential for systemic accumulation, and immunogenicity require further scrutiny, particularly in the context of repeated or sustained biomedical use [19,39,43,44,45]. While existing studies indicate that kaolinite exhibits low cytotoxicity and minimal pro-inflammatory responses under controlled conditions, especially when appropriately surface-modified, the possibility of bioaccumulation in reticuloendothelial organs (e.g., liver, spleen) and unintended immune activation cannot be fully excluded [19,39,43,44,45]. Therefore, comprehensive in vivo investigations addressing pharmacokinetics, long-term biodistribution, chronic toxicity, and potential immunogenic effects are essential to support the translational viability of kaolinite-based DDSs in biomedical applications.

## 5. Conclusions

This study introduces the innovative development of engineered phytocarrier systems derived from *Z. officinale* through the integration of kaolinite, resulting in the formation of the ZO–kaolinite system. The successful preparation of this novel carrier system was rigorously validated using a range of advanced analytical techniques, including FTIR spectroscopy, SEM, XRD, and DLS, confirming the system’s structural stability and integrity. Additionally, the micro-spray encapsulation of both the *Z. officinale* extract and the ZO–kaolinite system within a maltodextrin matrix led to the creation of two new phytocarrier systems: MZO and MZO–kaolinite. Characterization of these systems (using the same suite of techniques) revealed consistent structural properties across both systems. Thermal stability testing demonstrated that both systems retained their integrity under varying temperature conditions, which is essential for their application in diverse environmental settings. Biological evaluations indicated that the ZO–kaolinite and MZO–kaolinite systems exhibited significantly enhanced antioxidant, antimicrobial, and cytotoxic activities compared to the unencapsulated *Z. officinale* extract, underscoring their potential for more effective therapeutic applications. These results emphasize the crucial role of formulation strategies in enhancing the anticancer potential of bioactive compounds derived from *Z. officinale*. Moreover, this study establishes a strong foundation for future research focused on optimizing and standardizing kaolinite-based DDSs. Targeted quantification of key phytomarkers, such as gingerols and shogaols, will be critical for accurately evaluating encapsulation efficiency, improving batch-to-batch reproducibility, and enabling precise dose standardization. Future work will also prioritize detailed in vitro release kinetics studies to elucidate release mechanisms and bioavailability, alongside comprehensive investigations of formulation stability under varying environmental conditions (temperature, humidity, light exposure, etc.) to validate storage shelf-life and long-term biological performance. Furthermore, in vivo evaluations of the therapeutic efficacy and safety of these kaolinite-based phytocarriers in phytochemical-mediated cancer therapies will be essential to enhance clinical outcomes while minimizing systemic toxicity. Collectively, these findings highlight the significant potential of advanced phytocarrier systems to improve the delivery and biological effectiveness of plant-derived bioactive compounds in biomedical applications.

## Figures and Tables

**Table 1:** Main compounds identified by GC–MS analysis of the *Z. officinale* sample.

No.	t_R_ (min)	RI Determined	Kováts Index	Compound	Formula	Molecular Weight (g/mol)	Area (%)	Ref.
1	4.78	998	1000	octanal	C_8_H_16_O	128.21	1.71	[64,65]
2	5.43	2444	2445	isooctyl phthalate	C_24_H_38_O_4_	390.56	1.83	[64,65]
3	6.62	1174	1175	decanal	C_10_H_20_O	156.26	2.08	[64,65]
4	7.09	1341	1343	eugenol	C_10_H_12_O_2_	164.20	1.11	[64,65]
5	8.22	796	798	hexanal	C_6_H_12_O	100.16	1.23	[64,65]
6	8.88	1036	1039	cineole	C_10_H_18_O	154.25	1.83	[64,65]
7	9.54	2656	2657	squalene	C_30_H_50_	410.7	1.77	[64,65]
8	10.01	1408	1410	vanillin	C_8_H_8_O_3_	152.15	1.42	[64,65]
9	10.94	1177	1179	α-terpinene	C_10_H_16_	136.23	1.57	[64,65,66,67]
10	11.15	1238	1240	citral	C_10_H_16_O	152.23	2.62	[64,65,66,67]
11	12.09	1493	1496	zingiberene	C_15_H_24_	204.35	10.18	[67]
12	13.67	1656	1657	zingiberol	C_15_H_26_O	222.37	5.07	[64,65,66,67]
13	14.91	1323	1385	linalool	C_10_H_18_O	154.25	3.39	[64,65]
14	15.73	1387	1389	2-nonanone	C_9_H_18_O	142.24	3.12	[64,65]
15	16.42	981	983	methylheptenone	C_8_H_14_O	126.20	2.62	[64,65]
16	17.93	1354	1356	citronellyl acetate	C_12_H_22_O_2_	198.30	3.23	[64,65]
17	19.08	1093	1095	camphor	C_10_H_16_O	152.23	1.62	[64,65]
18	19.62	1152	1154	myrtenal	C_10_H_14_O	150.22	1.16	[64,65]
19	20.17	2182	2184	gingerol	C_17_H_26_O_4_	294.4	5.44	[64,65]
20	20.96	972	974	2-heptanone	C_7_H_14_O	114.19	2.03	[64,65]
21	21.36	1485	1487	curcumene	C_15_H_22_	202.33	2.57	[64,65,66,67]
22	22.21	1276	1277	bornyl acetate	C_12_H_20_O_2_	196.29	4.36	[64,65]
23	23.07	1345	1347	ethyl cinnamate	C_11_H_12_O_2_	176.21	1.73	[64,65]
24	24.03	1279	1281	ascaridole	C_10_H_16_O_2_	168.23	2.17	[64,65]
25	24.77	597	599	ethyl acetate	C_4_H_8_O_2_	88.11	1.78	[64,65,66]
26	25.68	1650	1651	zingerone	C_11_H_14_O_3_	194.23	5.42	[64,65,66,67]
27	26.93	2085	2087	4-shogaol	C_15_H_20_O_3_	248.32	4.26	[67]
28	27.76	2234	2235	6-paradol	C_17_H_26_O_3_	278.40	3.51	[67]
29	29.11	1167	1168	decanone	C_10_H_20_O	156.26	1.53	[64,65]
30	30.81	1498	1451	α-cadinene	C_15_H_26_	204.35	1.49	[64,65]
31	32.22	1296	1297	2-undecanone	C_11_H_22_O	170.29	2.06	[64,65]
32	34.52	1579	1581	spathulenol	C_15_H_24_O	220.35	1.23	[64,65]
33	38.86	1732	1734	zerumbone	C_15_H_22_O	218.33	3.12	[67]

GC–MS: gas chromatography–mass spectrometry; RI: retention index; t_R_: retention time.

**Table 2:** Phytochemicals identified by MS analysis of the *Z. officinale* sample.

No.	*m/z* Detected	Theoretic *m/z*	Formula	Tentative Identification	Category	Ref.
1	76.07	75.07	C_2_H_5_NO_2_	glycine	amino acids	[7,10,68,69]
2	90.09	89.09	C_3_H_7_NO_2_	alanine	amino acids	[7,10,68,69]
3	106.09	105.09	C_3_H_7_NO_3_	serine	amino acids	[7,10,68,69]
4	116.14	115.13	C_5_H_9_NO_2_	proline	amino acids	[7,10,68,69]
5	118.15	117.15	C_5_H_11_NO_2_	valine	amino acids	[7,10,68,69]
6	120.12	119.12	C_4_H_9_NO_3_	threonine	amino acids	[7,10,68,69]
7	122.16	121.16	C_3_H_7_NO_2_S	cysteine	amino acids	[7,10,68,69]
8	132.17	131.17	C_6_H_13_NO_2_	leucine	amino acids	[7,10,68,69]
9	134.11	133.10	C_4_H_7_NO_4_	aspartic acid	amino acids	[7,10,26,68,69]
10	147.11	146.12	C_5_H_8_NO_4_	glutamate	amino acids	[7,10,68,69]
11	147.18	146.19	C_6_H_14_N_2_O_2_	lysine	amino acids	[7,10,68,69]
12	150.22	149.21	C_5_H_11_NO_2_S	methionine	amino acids	[7,10,68,69]
13	156.14	155.15	C_6_H_9_N_3_O_2_	histidine	amino acids	[7,10,68,69]
14	166.18	165.19	C_9_H_11_NO_2_	phenylalanine	amino acids	[7,10,68,69]
15	175.21	174.20	C_6_H_14_N_4_O_2_	arginine	amino acids	[7,10,68,69]
16	182.18	181.19	C_9_H_11_NO_3_	tyrosine	amino acids	[7,10,68,69]
17	205.23	204.22	C_11_H_12_N_2_O_2_	tryptophan	amino acids	[7,10,68,69]
18	207.25	206.24	C_12_H_14_O_3_	eugenyl acetate	phenylpropanoids	[7,10,68,69]
19	357.41	356.40	C_21_H_24_O_5_	gingerenone A	diarylheptanoids	[7,10,14,23,24,25,26,27,28,68,69]
20	375.39	374.40	C_21_H_26_O_6_	hexahydrocurcumin	diarylheptanoids	[7,10,14,23,24,25,26,27,28,68,69]
21	381.49	380.48	C_21_H_32_O_6_	diacetoxy-6-gingerdiol	diarylheptanoids	[7,10,14,23,24,25,26,27,28,68,69]
22	401.41	400.40	C_21_H_20_O_8_	dihydroxy-curcumin	diarylheptanoids	[7,10,14,23,24,25,26,27,28,68,69]
23	291.39	290.40	C_17_H_22_O_4_	6-dehydro-gingerdione	gingerols (polyphenols)	[7,10,14,23,24,25,26,27,28,68,69]
24	145.22	144.21	C_8_H_16_O_2_	caprylic acid	fatty acids	[7,10,68,69,70]
25	173.26	172.26	C_10_H_20_O_2_	capric acid	fatty acids	[7,10,68,69,70]
26	201.31	200.32	C_12_H_24_O_2_	lauric acid	fatty acids	[7,10,68,69,70]
27	229.37	228.37	C_14_H_28_O_2_	myristic acid	fatty acids	[7,10,68,69,70]
28	255.41	254.41	C_16_H_30_O_2_	palmitoleic acid	fatty acids	[7,10,68,69,70]
29	271.49	270.50	C_17_H_34_O_2_	margaric acid	fatty acids	[7,10,68,69,70]
30	285.51	284.50	C_18_H_36_O_2_	stearic acid	fatty acids	[7,10,68,69,70]
31	313.49	312.50	C_20_H_40_O_2_	arachidic acid	fatty acids	[7,10,68,69,70]
32	341.59	340.60	C_22_H_44_O_2_	behenic acid	fatty acids	[7,10,68,69,70]
33	369.61	368.60	C_24_H_48_O_2_	lignoceric acid	fatty acids	[7,10,68,69,70]
34	187.29	186.29	C_11_H_22_O_2_	undecanoic acid	fatty acids	[7,10,68,69,70]
35	257.43	256.42	C_16_H_32_O_2_	palmitic acid	fatty acids	[7,10,68,69,70]
36	281.39	280.40	C_18_H_32_O_2_	linoleic acid	fatty acids	[7,10,68,69,70]
37	283.51	282.50	C_18_H_34_O_2_	oleic acid	fatty acids	[7,10,68,69,70]
38	271.25	270.24	C_15_H_10_O_5_	apigenin	flavonoids	[7,10,25,68,69]
39	287.23	286.24	C_15_H_10_O_6_	kaempferol	flavonoids	[7,10,25,68,69]
40	291.27	290.27	C_15_H_14_O_6_	catechin	flavonoids	[7,10,25,68,69]
41	303.24	302.23	C_15_H_10_O_7_	quercetin	flavonoids	[7,10,25,68,69]
42	611.49	610.50	C_27_H_30_O_16_	rutin	flavonoids	[7,10,25,68,69]
43	171.11	170.12	C_7_H_6_O_5_	gallic acid	phenolic acids	[7,10,25,68,69]
44	195.17	194.18	C_10_H_10_O_4_	ferulic acid	phenolic acids	[7,10,25,68,69]
45	149.17	148.16	C_9_H_8_O_2_	cinnamic acid	phenolic acids	[7,10,25,68,69]
46	139.13	138.12	C_7_H_6_O_3_	salicylic acid	phenolic acids	[7,10,25,68,69]
47	361.31	360.30	C_18_H_16_O_8_	rosmarinic acid	phenolic acids	[7,10,25,68,69]
48	155.12	154.12	C_7_H_6_O_4_	protocatechuic acid	phenolic acids	[7,10,25,68,69]
49	413.69	412.70	C_29_H_48_O	stigmasterol	sterols	[7,10,68,69]
50	415.71	414.70	C_29_H_50_O	β-sitosterol	sterols	[7,10,68,69]
51	577.81	576.80	C_35_H_60_O_6_	daucosterol	sterols	[7,10,68,69]
52	137.23	136.23	C_10_H_16_	α-terpinene	terpenes	[7,10,12,13,14,23,24,25,26,27,28,68,69,70,71,72]
53	151.21	150.22	C_10_H_14_O	myrtenal	terpenes	[7,10,12,13,14,23,24,25,26,27,28,68,69,70,71,72]
54	153.23	152.23	C_10_H_16_O	citral	terpenes	[7,10,12,13,14,23,24,25,26,27,28,68,69,70,71,72]
55	155.25	154.25	C_10_H_18_O	cineole	terpenes	[7,10,12,13,14,23,24,25,26,27,28,68,69,70,71,72]
56	155.25	154.25	C_10_H_18_O	linalool	terpenes	[7,10,12,13,14,23,24,25,26,27,28,68,69,70,71,72]
57	165.19	164.20	C_10_H_12_O_2_	eugenol	terpenes	[7,10,12,13,14,23,24,25,26,27,28,68,69,70,71,72]
58	169.23	168.23	C_10_H_16_O_2_	ascaridole	terpenes	[7,10,12,13,14,23,24,25,26,27,28,68,69,70,71,72]
59	171.24	170.25	C_10_H_18_O_2_	8-hydroxy geraniol	terpenes	[7,10,12,13,14,23,24,25,26,27,28,68,69,70,71,72]
60	197.29	196.29	C_12_H_20_O_2_	bornyl acetate	terpenes	[7,10,12,13,14,23,24,25,26,27,28,68,69,70,71,72]
61	199.31	198.30	C_12_H_22_O_2_	citronellyl acetate	terpenes	[7,10,12,13,14,23,24,25,26,27,28,68,69,70,71,72]
62	203.32	202.33	C_15_H_22_	α-curcumene	terpenes	[7,10,12,13,14,23,24,25,26,27,28,68,69,70,71,72]
63	205.35	204.35	C_15_H_24_	zingiberene	terpenes	[7,10,12,13,14,23,24,25,26,27,28,68,69,70,71,72]
64	207.36	206.37	C_15_H_26_	cadinene	terpenes	[7,10,12,13,14,23,24,25,26,27,28,68,69,70,71,72]
65	219.34	218.33	C_15_H_22_O	zerumbone	terpenes	[7,10,12,13,14,23,24,25,26,27,28,68,69,70,71,72]
66	221.35	220.35	C_15_H_24_O	spathulenol	terpenes	[7,10,12,13,14,23,24,25,26,27,28,68,69,70,71,72]
67	223.37	222.37	C_15_H_26_O	zingiberol	terpenes	[7,10,12,13,14,23,24,25,26,27,28,68,69,70,71,72]
68	409.51	408.50	C_22_H_32_O_7_	ingol-12-acetate	terpenes	[7,10,12,13,14,23,24,25,26,27,28,68,69,70,71,72]
69	411.69	410.70	C_30_H_50_	squalene	terpenes	[7,10,12,13,14,23,24,25,26,27,28,68,69,70,71,72]
70	151.18	150.17	C_9_H_10_O_2_	4-vinylguaiacol	phenols	[7,10,68,69]
71	295.41	294.40	C_17_H_26_O_4_	gingerol	gingerols (polyphenols)	[7,10,12,13,14,23,24,25,26,27,28,68,69,70,71,72]
72	291.39	290.40	C_17_H_22_O_4_	dehydrogingerdione	gingerols (polyphenols)	[7,10,12,13,14,23,24,25,26,27,28,68,69,70,71,72,73]
73	295.41	294.40	C_17_H_26_O_4_	6-gingerol	gingerols (polyphenols)	[7,10,12,13,14,23,24,25,26,27,28,68,69,70,71,72]
74	309.39	308.40	C_18_H_28_O_4_	methyl-6-gingerol	gingerols (polyphenols)	[7,10,12,13,14,23,24,25,26,27,28,68,69,70,71,72]
75	323.45	322.44	C_19_H_30_O_4_	8-gingerol	gingerols (polyphenols)	[7,10,12,13,14,23,24,25,26,27,28,68,69,70,71,72]
76	351.49	350.50	C_21_H_34_O_4_	10-gingerol	gingerols (polyphenols)	[7,10,12,13,14,23,24,25,26,27,28,68,69,70,71,72]
77	381.51	380.50	C_21_H_32_O_6_	diacetoxy-6-gingerdiol	gingerols (polyphenols)	[7,10,12,13,14,23,24,25,26,27,28,68,69,70,71,72]
78	393.61	392.60	C_24_H_40_O_4_	methyl-12-gingerol	gingerols (polyphenols)	[7,10,12,13,14,23,24,25,26,27,28,68,69,70,71,72]
79	359.49	358.50	C_17_H_26_O_6_S	6-gingesulfonic acid	sulfonates	[7,10,12,13,14,23,24,25,26,27,28,68,69,70,71,72]
80	297.39	296.40	C_17_H_28_O_4_	gingerdiol	fatty alcohol esters	[7,10,12,13,14,23,24,25,26,27,28,68,69,70,71,72]
81	249.33	248.32	C_15_H_20_O_3_	4-shogaol	shogaols (polyphenols)	[7,10,12,13,14,23,24,25,26,27,28,68,69,70,71,72]
82	275.35	274.35	C_17_H_22_O_3_	6-dehydroshogaol	shogaols (polyphenols)	[7,10,12,13,14,23,24,25,26,27,28,68,69,70,71,72,73]
83	277.39	276.40	C_17_H_24_O_3_	shogaol	shogaols (polyphenols)	[7,10,12,13,14,23,24,25,26,27,28,68,69,70,71,72]
84	333.51	332.50	C_21_H_32_O_3_	10-shogaol	shogaols (polyphenols)	[7,10,12,13,14,23,24,25,26,27,28,68,69,70,71,72]
85	361.54	360.53	C_23_H_36_O_3_	12-shogaol	gingerols (polyphenols)	[7,10,12,13,14,23,24,25,26,27,28,68,69,70,71,72]
86	279.39	278.40	C_17_H_26_O_3_	paradol	paradols (polyphenols)	[7,10,12,13,14,23,24,25,26,27,28,68,69,70,71,72]
87	307.45	306.44	C_19_H_30_O_3_	8-paradol	paradols (polyphenols)	[7,10,12,13,14,23,24,25,26,27,28,68,69,70,71,72]
88	89.12	88.11	C_4_H_8_O_2_	ethyl acetate	esters	[7,10,12,13,14,23,24,25,26,27,28,68,69,70,71,72]
89	177.21	176.21	C_11_H_12_O_2_	ethyl cinnamate	esters	[7,10,12,13,14,23,24,25,26,27,28,68,69,70,71,72]
90	391.61	390.60	C_24_H_38_O_4_	isooctyl phthalate	esters	[7,10,12,13,14,23,24,25,26,27,28,68,69,70,71,72]
91	338.59	337.60	C_22_H_43_NO	13-docosamide	amides	[7,10,12,13,14,23,24,25,26,27,28,68,69,70,71,72]
92	153.15	152.15	C_8_H_8_O_3_	vanillin	aldehydes	[7,10,12,13,14,23,24,25,26,27,28,68,69,70,71,72]
93	157.25	156.26	C_10_H_20_O	decanal	aldehydes	[7,10,12,13,14,23,24,25,26,27,28,68,69,70,71,72]
94	101.17	100.16	C_6_H_12_O	hexanal	aldehydes	[7,10,12,13,14,23,24,25,26,27,28,68,69,70,71,72]
95	129.21	128.21	C_8_H_16_O	octanal	aldehydes	[7,10,12,13,14,23,24,25,26,27,28,68,69,70,71,72]
96	115.19	114.19	C_7_H_14_O	2-heptanone	ketones	[7,10,12,13,14,23,24,25,26,27,28,68,69,70,71,72]
97	127.21	126.20	C_8_H_14_O	methylheptenone	ketones	[7,10,12,13,14,23,24,25,26,27,28,68,69,70,71,72]
98	143.25	142.24	C_9_H_18_O	2-nonanone	ketones	[7,10,12,13,14,23,24,25,26,27,28,68,69,70,71,72]
99	157.25	156.26	C_10_H_20_O	decanone	ketones	[7,10,12,13,14,23,24,25,26,27,28,68,69,70,71,72]
100	171.29	170.29	C_11_H_22_O	2-undecanone	ketones	[7,10,12,13,14,23,24,25,26,27,28,68,69,70,71,72]
101	195.23	194.23	C_11_H_14_O_3_	zingerone	ketones	[7,10,12,13,14,23,24,25,26,27,28,68,69,70,71,72]
102	43.09	42.08	C_3_H_6_	cyclopropane	hydrocarbons	[7,10,12,13,14,23,24,25,26,27,28,68,69,70,71,72]
103	85.17	84.16	C_6_H_12_	cyclohexane	hydrocarbons	[7,10,12,13,14,23,24,25,26,27,28,68,69,70,71,72]
104	349.59	348.60	C_25_H_48_	1-pentadecyl-decahydronaphthalene	hydrocarbons	[7,10,12,13,14,23,24,25,26,27,28,68,69,70,71,72]
105	367.71	366.70	C_26_H_54_	hexacosane	hydrocarbons	[7,10,12,13,14,23,24,25,26,27,28,68,69,70,71,72]

MS: mass spectrometry.

**Table 3:** Identification of VOCs in the *Z. officinale* sample through MS.

VOC	Odor Profile
α-terpinene	woody
myrtenal	spicy
citral	citrus
cineole	eucalyptus
eugenol	spicy
ascaridole	pungent
8-hydroxy geraniol	citrus
bornyl acetate	piney
citronellyl acetate	fruity
α-curcumene	spicy
zingiberene	spicy
cadinene	citrus
zerumbone	pungent
spathulenol	earthy
myrtenal	spicy
linalool	floral, woody
4-vinylguaiacol	spicy
ethyl acetate	fruity
ethyl cinnamate	fruity
vanillin	vanilla
decanal	citrus
hexanal	herbal
octanal	citrus
2-heptanone	fruity
methylheptenone	fruity
2-nonanone	cheesy
decanone	citrus
2-undecanone	fruity
zingerone	spicy
cyclopropane	petroleum
cyclohexane	sweet

MS: mass spectrometry; VOC: volatile organic compound.

**Table 4:** Characteristic vibrational peaks associated with phytochemicals from the *Z. officinale* sample.

Phytochemicals Category	Wavenumber (cm^−1^)	Ref.
terpenoids	2925; 2349; 1738; 1249; 1090; 998; 815	[7,74]
alkaloids	3388; 1652; 1639; 1602; 1569; 1405; 753; 738; 663	[7,74]
coumarins	3430; 1727; 1607; 1265; 1132; 1252; 900–603	[7,75]
flavonoids	3405–3100; 1526; 1460; 1438; 1363; 1270	[7,58]
phenolic acids	1364; 1250; 1240; 1171–1100; 1034	[57,58,74]
fatty acids	2925; 1351; 1247; 1089; 742; 720	[7,58,60]
phytosterols	1465; 1378; 1059; 741	[7,58,60]
phenylpropanoids	3190; 3003; 1635; 1505; 1451; 1246	[7,76,77]
phytoecdysteroids	3303; 1651	[7,76,77]
diarylheptanoids	2102–2549; 1621–1742	[77]
gingerols	3398; 2838; 1665; 1623; 1518; 1368; 1274; 1234; 1037; 962; 812	[7,76,77,78,79]
shogaols	2834; 1732; 1597; 1481; 1246; 1182; 815	[7,76,77,78,79]
amino acids	3401; 3328–3132; 2531–2758; 2128; 1725–1756; 1690; 1673; 1661; 1650; 1643; 1634; 1619; 1609; 1612–1658; 1502–1602	[58,59,61]
paradols	3402; 2970; 1601; 1298; 778	[7,76,77,78,79]
fatty acid esters	1745; 1310; 3450; 2972; 1469; 813	[80,81]

**Table 5:** Particle diameter distribution of the MZO carrier and the MZO–kaolinite system microcapsules.

Sample	Particle Size Diameter (μm)						Volume Diameter (μm)		
	D [3,2]			D [4,3]			d_10_	d_50_	d_90_
MZO carrier	771.21 ± 0.004			806.12 ± 0.002			431.13 ± 0.008	628.19 ± 0.012	879.23 ± 0.019
MZO–kaolinite system	Peak 1	Peak 2	Overall	Peak 1	Peak 2	Overall	4.14 ± 0.002	10.11 ± 0.013	90.41 ± 0.007
	8.42 ± 0.003	203.04 ± 0.002	34.27 ± 0.006	10.89 ± 0.005	251.73 ± 0.005	64.45 ± 0.004			

D [3,2] represents the surface-weighted mean diameter and D [4,3] represents the volume-weighted mean diameter. d_10_, d_50_, and d_90_ correspond to cumulative distributions at 10%, 50%, and 90%, respectively. MZO carrier: maltodextrin–*Z. officinale* carrier; MZO–kaolinite: maltodextrin–*Z. officinale*–kaolinite system.

**Table 6:** Encapsulation parameters for the prepared carriers.

Sample	EE%	EC%	EY%
MZO carrier	64.54 ± 0.12	62.23 ± 0.19	63.61 ± 0.17
MZO–kaolinite system	62.61 ± 0.09	60.05 ± 0.22	62.35 ± 0.34

EC%: loading capacity; EE%: encapsulation efficiency; EY%: encapsulation yield; MZO carrier: maltodextrin–*Z. officinale* carrier; MZO–kaolinite: maltodextrin–*Z. officinale*–kaolinite system.

**Table 7:** Antibacterial activity results against selected pathogenic microorganisms.

Pathogenic Microorganism	Sample	Inhibition Zone Diameter (mm)						
		Sample Concentration (μg/mL)					Positive Control (Gentamicin, 100 μg/mL)	Negative Control (DMSO)
		100	125	150	175	200		
*Staphylococcus aureus*	*Z. officinale*	24.02 ± 0.18	35.72 ± 0.57	43.13 ± 0.25	49.73 ± 0.33	56.84 ± 0.33	22.18 ± 0.22	0
	Kaolinite	12.35 ± 0.18	14.78 ± 0.23	16.17 ± 0.21	18.54 ± 0.36	20.87 ± 0.43		
	ZO–kaolinite	31.17 ± 0.31	43.32 ± 0.25	59.67 ± 0.34	67.73 ± 0.23	73.33 ± 0.41		
	MZO carrier	26.42 ± 0.16	37.93 ± 0.51	45.31 ± 0.26	51.34 ± 0.18	58.15 ± 0.63		
	MZO–kaolinite system	41.15 ± 0.41	47.68 ± 0.16	62.25 ± 0.17	72.31 ± 0.61	77.26 ± 0.58		
*Enterococcus faecalis*	*Z. officinale*	14.29 ± 0.13	16.55 ± 0.21	19.31 ± 0.32	21.07 ± 0.18	23.73 ± 0.08	24.31 ± 0.21	0
	Kaolinite	11.04 ± 0.15	12.89 ± 0.24	14.18 ± 0.31	16.27 ± 0.13	19.05 ± 0.42		
	ZO–kaolinite	18.70 ± 0.21	22.07 ± 0.32	29.44 ± 0.45	34.08 ± 0.22	40.13 ± 0.32		
	MZO carrier	16.77 ± 0.41	18.62 ± 0.17	21.19 ± 0.18	24.06 ± 0.22	27.05 ± 0.34		
	MZO–kaolinite system	27.36 ± 0.19	32.04 ± 0.13	38.22 ± 0.33	42.87 ± 0.54	45.47 ± 0.43		
*Bacillus cereus*	*Z. officinale*	13.27 ± 0.19	17.01 ± 0.08	21.32 ± 0.42	26.03 ± 0.33	29.85 ± 0.51	18.22 ± 0.16	0
	Kaolinite	10.49 ± 0.31	13.58 ± 0.28	17.05 ± 0.09	19.98 ± 0.17	22.04 ± 0.31		
	ZO–kaolinite	19.23 ± 0.16	20.61 ± 0.07	24.18 ± 0.22	34.91 ± 0.46	44.77 ± 0.33		
	MZO carrier	15.18 ± 0.28	19.13 ± 0.11	22.86 ± 0.08	28.54 ± 0.42	31.39 ± 0.47		
	MZO–kaolinite system	24.38 ± 0.11	28.03 ± 0.63	32.68 ± 0.35	39.89 ± 0.17	48.06 ± 0.27		
*Pseudomonas aeruginosa*	*Z. officinale*	30.03 ± 0.15	35.12 ± 0.21	41.14 ± 0.35	49.26 ± 0.07	53.47 ± 0.32	30.54 ± 0.21	0
	Kaolinite	13.08 ± 0.09	19.22 ± 0.12	24.66 ± 0.23	29.88 ± 0.48	35.62 ± 0.51		
	ZO–kaolinite	35.25 ± 0.21	49.87 ± 0.36	57.28 ± 0.51	62.82 ± 0.08	69.02 ± 0.38		
	MZO carrier	32.18 ± 0.11	37.14 ± 0.23	43.32 ± 0.34	51.18 ± 0.22	55.02 ± 0.41		
	MZO–kaolinite system	40.29 ± 0.63	56.37 ± 0.42	62.87 ± 0.17	69.27 ± 0.26	74.95 ± 0.14		
*Klebsiella pneumoniae*	*Z. officinale*	40.02 ± 0.31	48.65 ± 0.23	54.22 ± 0.27	59.32 ± 0.24	66.47 ± 0.18	15.75 ± 0.23	0
	Kaolinite	10.51 ± 0.06	13.23 ± 0.22	16.99 ± 0.13	19.89 ± 0.42	21.78 ± 0.33		
	ZO–kaolinite	42.34 ± 0.09	50.76 ± 0.34	56.43 ± 0.23	70.12 ± 0.17	78.72 ± 0.42		
	MZO carrier	49.67 ± 0.34	50.52 ± 0.51	56.23 ± 0.61	61.85 ± 0.17	68.75 ± 0.26		
	MZO–kaolinite system	58.89 ± 0.16	59.45 ± 0.35	65.27 ± 0.42	73.78 ± 0.23	82.03 ± 0.32		
*Escherichia coli*	*Z. officinale*	13.25 ± 0.31	15.61 ± 0.12	17.82 ± 0.27	19.94 ± 0.33	21.12 ± 0.07	20.59 ± 0.32	0
	Kaolinite	15.01 ± 0.67	18.89 ± 0.52	22.18 ± 0.27	26.87 ± 0.35	31.22 ± 0.18		
	ZO–kaolinite	21.26 ± 0.31	28.76 ± 0.23	30.66 ± 0.37	39.98 ± 0.42	43.02 ± 0.23		
	MZO carrier	15.75 ± 0.22	17.64 ± 0.41	19.78 ± 0.53	21.58 ± 0.33	23.85 ± 0.16		
	MZO–kaolinite system	26.72 ± 0.13	35.54 ± 0.32	40.16 ± 0.23	47.21 ± 0.43	50.63 ± 0.52		

Values are expressed as the mean ± SD (*n* = 3). DMSO: dimethyl sulfoxide; SD: standard deviation.

**Table 8:** MICs and MBCs of samples against selected pathogenic microorganisms.

Pathogenic Microorganism	Sample	MIC (μg/mL)	MBC (μg/mL)	Gentamicin	
				MIC (μg/mL)	MBC (μg/mL)
*Staphylococcus aureus*	*Z. officinale*	0.73 ± 0.09	0.74 ± 0.11	0.62 ± 0.45	0.62 ± 0.49
	Kaolinite	1.22 ± 0.26	1.41 ± 0.09		
	ZO–kaolinite	0.43 ± 0.05	0.45 ± 0.19		
	MZO carrier	0.65 ± 0.01	0.66 ± 0.13		
	MZO–kaolinite system	0.35 ± 0.07	0.32 ± 0.03		
*Enterococcus faecalis*	*Z. officinale*	0.57 ± 0.19	0.56 ± 0.11	0.49 ± 0.14	0.45 ± 0.19
	Kaolinite	0.71 ± 0.07	0.69 ± 0.13		
	ZO–kaolinite	0.42 ± 0.09	0.43 ± 0.04		
	MZO carrier	0.51 ± 0.07	0.52 ± 0.15		
	MZO–kaolinite system	0.29 ± 0.05	0.30 ± 0.11		
*Bacillus cereus*	*Z. officinale*	1.32 ± 0.11	1.34 ± 0.17	1.29 ± 0.05	1.28 ± 0.03
	Kaolinite	0.87 ± 0.31	0.89 ± 0.29		
	ZO–kaolinite	0.29 ± 0.24	0.28 ± 0.41		
	MZO carrier	1.28 ± 0.22	1.27 ± 0.11		
	MZO–kaolinite system	0.26 ± 0.09	0.25 ± 0.05		
*Pseudomonas aeruginosa*	*Z. officinale*	1.73 ± 0.08	1.75 ± 0.53	1.97 ± 0.32	1.96 ± 0.26
	Kaolinite	1.91 ± 0.21	1.90 ± 0.35		
	ZO–kaolinite	1.34 ± 0.12	1.41 ± 0.43		
	MZO carrier	1.71 ± 0.02	1.72 ± 0.09		
	MZO–kaolinite system	1.32 ± 0.13	1.31 ± 0.16		
*Klebsiella pneumoniae*	*Z. officinale*	0.79 ± 0.12	0.79 ± 0.17	0.82 ± 0.19	0.82 ± 0.17
	Kaolinite	1.02 ± 0.14	1.01 ± 0.33		
	ZO–kaolinite	0.66 ± 0.23	0.66 ± 0.21		
	MZO carrier	0.69 ± 0.17	0.71 ± 0.15		
	MZO–kaolinite system	0.61 ± 0.08	0.63 ± 0.03		
*Escherichia coli*	*Z. officinale*	1.14 ± 0.13	1.15 ± 0.08	1.11 ± 0.25	1.11 ± 0.31
	Kaolinite	1.38 ± 0.65	1.61 ± 0.42		
	ZO–kaolinite	0.98 ± 0.31	0.96 ± 0.07		
	MZO carrier	1.12 ± 0.04	1.12 ± 0.13		
	MZO–kaolinite system	0.83 ± 0.21	0.85 ± 0.03		

Values are expressed as the mean ± SD (*n* = 3). MBC: minimum bactericidal concentration; MIC: minimum inhibitory concentration; SD: standard deviation.

## Data Availability

The original contributions presented in this study are included in this article. Further inquiries can be directed to the corresponding author.

## Abbreviations

The following abbreviations are used in this manuscript:

AAEs	Ascorbic acid equivalents
Akt/mTOR	Protein kinase B/mammalian target of rapamycin
AMR	Antimicrobial resistance
ANOVA	Analysis of variance
ASTM	American Society for Testing and Materials
ATCC	American Type Culture Collection
ATR	Attenuated total reflectance
CFU	Colony-forming units
CRKP	Carbapenem-resistant *K. pneumoniae*
DDS	Drug delivery system
ΔH	Enthalpy change
DLS	Dynamic light scattering
DMEM	Dulbecco’s Modified Eagle’s Medium
DMSO	Dimethyl sulfoxide
DNA	Deoxyribonucleic acid
DPPH	2,2-Diphenyl-1-picrylhydrazyl
DTA	Differential thermal analysis
DTG	Derivative thermogravimetry
EC%	Loading capacity
EDS	Energy-dispersive X-ray (EDX) spectroscopy
EE%	Encapsulation efficiency
ESI–QTOF–MS	Electro-spray ionization–quadrupole time-of-flight–mass spectrometry
EHEC	Enterohemorrhagic *E. coli*
ETEC	Enterotoxigenic *E. coli*
EY%	Encapsulation yield
FBS	Fetal bovine serum
FEG	Field emission gun
FRAP	Ferric reducing antioxidant power
FTIR	Fourier-transform infrared
GAE	Gallic acid equivalents
GC–MS	Gas chromatography–mass spectrometry
GRAS	Generally recognized as safe
HF	Heat flow
HT	High temperature
IC_50_	Half-maximal inhibitory concentration
ICDD	International Centre for Diffraction Data
IL-6	Interleukin-6
IS	Internal standard
IZ	Inhibition zone
MBC	Minimum bactericidal concentration
MIC	Minimum inhibitory concentration
MMP-9	Matrix metalloproteinase-9
MRSA	Methicillin-resistant *S. aureus*
MTT	3-(4,5-Dimethylthiazol-2-yl)-2,5-diphenyl tetrazolium bromide
MZO	Maltodextrin–*Zingiber officinale*
NBS	National Bureau of Standards
NF-κB	Nuclear factor-kappa B
NIST	National Institute of Standards and Technology
PDI	Polydispersity index
PSD	Particle size distribution
ROS	Reactive oxygen species
RT	Room temperature
SD	Standard deviation
SEM	Scanning electron microscopy
TAC	Total antioxidant capacity
TG	Thermogravimetry
TNF-α	Tumor necrosis factor-alpha
TPC	Total phenolic content
UAE	Ultrasound-assisted extraction
UPEC	Uropathogenic *E. coli*
UTI	Urinary tract infection
UV–Vis	Ultraviolet–visible
VOC	Volatile organic compound
VRE	Vancomycin resistance
WPPF	Whole powder profile fitting
XRD	X-ray diffraction
ZO	*Zingiber officinale*

## References

[1] Szymczak J., Grygiel-Górniak B., Cielecka-Piontek J. (2024). *Zingiber officinale* Roscoe: The Antiarthritic Potential of a Popular Spice—Preclinical and Clinical Evidence. Nutrients.

[2] Banerjee S., Banerjee J., Mullick H.I., Ghosh A.K. (2011). *Zingiber officinale*: A natural gold. Int. J. Pharm. Biosci..

[3] Stan D., Enciu A.M., Mateescu A.L., Ion A.C., Brezeanu A.C., Stan D., Tanase C. (2021). Natural Compounds with Antimicrobial and Antiviral Effect and Nanocarriers Used for Their Transportation. Front. Pharmacol..

[4] Fadaki F., Modaresi M., Sajjadian I. (2017). The Effects of Ginger Extract and Diazepam on Anxiety Reduction in Animal Model. Indian J. Pharm. Educ. Res..

[5] Bartels E.M., Folmer V.N., Bliddal H., Altman R.D., Juhl C., Tarp S., Zhang W., Christensen R. (2015). Efficacy and safety of ginger in osteoarthritis patients: A meta-analysis of randomized placebo-controlled trials. Osteoarthritis Cartil..

[6] Crichton M., Marshall S., Marx W., McCarthy A.L., Isenring E. (2019). Efficacy of Ginger (*Zingiber officinale*) in Ameliorating Chemotherapy-Induced Nausea and Vomiting and Chemotherapy-Related Outcomes: A Systematic Review Update and Meta-Analysis. J. Acad. Nutr. Diet..

[7] Mao Q.-Q., Xu X.-Y., Cao S.-Y., Gan R.-Y., Corke H., Beta T., Li H.-B. (2019). Bioactive Compounds and Bioactivities of Ginger (*Zingiber officinale* Roscoe). Foods.

[8] Balogun F.O., Tayo AdeyeOluwa E., Ashafa A.O.T., Wang H. (2020). Pharmacological potentials of ginger. Ginger Cultivation and Its Antimicrobial and Pharmacological Potentials.

[9] Shahrajabian M.H., Sun W., Cheng Q. (2019). Clinical aspects and health benefits of ginger (*Zingiber officinale*) in both traditional Chinese medicine and modern industry. Acta Agric. Scand. Sect. B Soil Plant Sci..

[10] Zhang M., Zhao R., Wang D., Wang L., Zhang Q., Wei S., Lu F., Peng W., Wu C. (2020). Ginger (*Zingiber officinale* Rosc.) and its bioactive components are potential resources for health beneficial agents. Phytother. Res..

[11] López E.I.C., Balcázar M.F.H., Mendoza J.M.R., Ortiz A.D.R., Melo M.T.O., Parrales R.S., Delgado T.H. (2017). Antimicrobial Activity of Essential Oil of *Zingiber officinale* Roscoe (*Zingiberaceae*). Am. J. Plant Sci..

[12] Momoh J.O., Olaleye O.N. (2022). Evaluation of Secondary Metabolites Profiling of Ginger (*Zingiber officinale* Roscoe) Rhizome using GC–MS and Its Antibacterial Potential on *Staphylococcus aureus* and *Escherichia coli*. Microbiol. Res. J. Int..

[13] Aleem M., Imran Khan M., Shakshaz F.A., Akbari N., Anwar D. (2020). Botany, phytochemistry and antimicrobial activity of ginger (*Zingiber officinale*): A review. Int. J. Herb. Med..

[14] Nalbantsoy A., Ayyildiz Tamiş D., Akgün I.H., Öztürk Yalçin T., Deliloğlu Gürhan I., Karaboz İ. (2008). Antimicrobial and Cytotoxic Activities of *Zingiber officinalis* Extracts. FABAD J. Pharm. Sci..

[15] Nath R., Roy R., Barai G., Bairagi S., Manna S., Chakraborty R. (2021). Modern Developments of Nano Based Drug Delivery System by Combined with Phytochemicals—Presenting New Aspects. Int. J. Sci. Res. Sci. Technol..

[16] Aparicio-Blanco J., Vishwakarma N., Lehr C.M., Prestidge C.A., Thomas N., Roberts R.J., Thorn C.R., Melero A. (2024). Antibiotic resistance and tolerance: What can drug delivery do against this global threat?. Drug Deliv. Transl. Res..

[17] Ahmed S.K., Hussein S., Qurbani K., Ibrahim R.H., Fareeq A., Mahmood K.A., Mohamed M.G. (2024). Antimicrobial resistance: Impacts, challenges, and future prospects. J. Med. Surg. Publ. Health.

[18] Singh I.P., Ahmad F., Chatterjee D., Bajpai R., Sengar N., Poduri R. (2021). Natural products: Drug discovery and development. Drug Discovery and Development: From Targets and Molecules to Medicines.

[19] Wu Q., Liao J., Yang H. (2023). Recent Advances in Kaolinite Nanoclay as Drug Carrier for Bioapplications: A Review. Adv. Sci. (Weinh.).

[20] Ferlay J., Colombet M., Soerjomataram I., Parkin D.M., Piñeros M., Znaor A., Bray F. (2021). Cancer statistics for the year 2020: An overview. Int. J. Cancer.

[21] Emran T.B., Shahriar A., Mahmud A.R., Rahman T., Abir M.H., Siddiquee M.F.R., Ahmed H., Rahman N., Nainu F., Wahyudin E. (2022). Multidrug Resistance in Cancer: Understanding Molecular Mechanisms, Immunoprevention and Therapeutic Approaches. Front. Oncol..

[22] Mei H., Cai S., Huang D., Gao H., Cao J., He B. (2022). Carrier-free nanodrugs with efficient drug delivery and release for cancer therapy: From intrinsic physicochemical properties to external modification. Bioact. Mater..

[23] Lee Y. (2016). Cytotoxicity Evaluation of Essential Oil and Its Component from *Zingiber officinale* Roscoe. Toxicol. Res..

[24] Mahomoodally M.F., Aumeeruddy M.Z., Rengasamy K.R.R., Roshan S., Hammad S., Pandohee J., Hu X., Zengin G. (2019). Ginger and its active compounds in cancer therapy: From folk uses to nano-therapeutic applications. Semin. Cancer Biol..

[25] Peng F., Tao Q., Wu X., Dou H., Spencer S., Mang C., Xu L., Sun L., Zhao Y., Li H. (2012). Cytotoxic, cytoprotective and antioxidant effects of isolated phenolic compounds from fresh ginger. Fitoterapia.

[26] Lechner J.F., Stoner G.D. (2019). Gingers and Their Purified Components as Cancer Chemopreventative Agents. Molecules.

[27] Mustafa I., Chin N.L. (2023). Antioxidant Properties of Dried Ginger (*Zingiber officinale* Roscoe) var. Bentong. Foods.

[28] Mošovská S., Nováková D., Kaliňák M. (2015). Antioxidant activity of ginger extract and identification of its active components. Acta Chim. Slovaca.

[29] Yang L., Wen K.-S., Ruan X., Zhao Y.-X., Wei F., Wang Q. (2018). Response of Plant Secondary Metabolites to Environmental Factors. Molecules.

[30] Segneanu A.E., Grozescu I., Sfirloaga P. (2013). The influence of extraction process parameters of some biomaterials precursors from *Helianthus annuus*. Dig. J. Nanomater. Biostruct..

[31] Popescu C., Fitigau F., Segneanu A.E., Balcu I., Martagiu R., Vaszilcsin C.G. (2011). Separation and characterization of anthocyanins by analytical and electrochemical methods. Environ. Eng. Manag. J..

[32] Vaszilcsin C.G., Segneanu A.E., Balcu I., Pop R., Fitigău F., Mirica M.C. (2010). Eco-friendly extraction and separation methods of capsaicines. Environ. Eng. Manag. J..

[33] Segneanu A.E., Damian D., Hulka I., Grozescu I., Salifoglou A. (2016). A simple and rapid method for calixarene-based selective extraction of bioactive molecules from natural products. Amino Acids.

[34] Segneanu A.E., Dabici A., Zuguang Y., Jianrong L., Grozescu I. (2012). Comparative analytical study of active compounds from *Zingiber officinale*. Optoelectron. Adv. Mater..

[35] Bejenaru L.E., Radu A., Segneanu A.-E., Biţă A., Manda C.-V., Mogoşanu G.D., Bejenaru C. (2024). Innovative Strategies for Upcycling Agricultural Residues and Their Various Pharmaceutical Applications. Plants.

[36] Dewi M.K., Chaerunisaa A.Y., Muhaimin M., Joni I.M. (2022). Improved Activity of Herbal Medicines through Nanotechnology. Nanomaterials.

[37] Stojiljković S.T., Stojiljković M.S. Application of bentonite clay for human use. Proceedings of the 4th Advanced Ceramics and Applications Conference.

[38] Williams L.B. (2019). Natural Antibacterial Clays: Historical Uses and Modern Advances. Clays Clay Miner..

[39] Massaro M., Colletti C.G., Lazzara G., Riela S. (2018). The Use of Some Clay Minerals as Natural Resources for Drug Carrier Applications. J. Funct. Biomater..

[40] Rebitski E.P., Darder M., Sainz-Diaz C.I., Carraro R., Aranda P., Ruiz-Hitzky E. (2020). Theoretical and experimental investigation on the intercalation of metformin into layered clay minerals. Appl. Clay Sci..

[41] Behroozian S., Zlosnik J.E.A., Xu W., Li L.Y., Davies J.E. (2023). Antibacterial Activity of a Natural Clay Mineral against *Burkholderia cepacia* Complex and Other Bacterial Pathogens Isolated from People with Cystic Fibrosis. Microorganisms.

[42] Martsouka F., Papagiannopoulos K., Hatziantoniou S., Barlog M., Lagiopoulos G., Tatoulis T., Tekerlekopoulou A.G., Lampropoulou P., Papoulis D. (2021). The Antimicrobial Properties of Modified Pharmaceutical Bentonite with Zinc and Copper. Pharmaceutics.

[43] Awad M.E., López-Galindo A., Setti M., El-Rahmany M.M., Iborra C.V. (2017). Kaolinite in pharmaceutics and biomedicine. Int. J. Pharm..

[44] Awad M.E., López-Galindo A., El-Rahmany M.M., El-Desoky H.M., Viseras C. (2017). Characterization of Egyptian kaolins for health-care uses. Appl. Clay. Sci..

[45] Saad H., Ayed A., Srasra M., Attia S., Srasra E., Charrier-El Bouhtoury F., Tabbene O., Oueslati W. (2022). New trends in clay-based nanohybrid applications: Essential oil encapsulation strategies to improve their biological activity. Nanoclay—Recent Advances, New Perspectives and Applications.

[46] Laureanti E.J.G., Paiva T.S., de Matos Jorge L.M., Jorge R.M.M. (2023). Microencapsulation of bioactive compound extracts using maltodextrin and gum Arabic by spray and freeze-drying techniques. Int. J. Biol. Macromol..

[47] Xu Y., Yan X., Zheng H., Li J., Wu X., Xu J., Zhen Z., Du C. (2024). The application of encapsulation technology in the food industry: Classifications, recent advances, and perspectives. Food Chem. X.

[48] Bejenaru C., Radu A., Segneanu A.-E., Biţă A., Ciocîlteu M.V., Mogoşanu G.D., Bradu I.A., Vlase T., Vlase G., Bejenaru L.E. (2024). Pharmaceutical Applications of Biomass Polymers: Review of Current Research and Perspectives. Polymers.

[49] Lucero M., Estell R., Tellez M., Fredrickson E. (2009). A retention index calculator simplifies identification of plant volatile organic compounds. Phytochem. Anal..

[50] McNaught A.D., Wilkinson A. (1997). Compendium of Chemical Terminology: IUPAC Recommendations.

[51] Vargas V., Saldarriaga S., Sánchez F.S., Cuellar L.N., Paladines G.M. (2024). Effects of the spray-drying process using maltodextrin on bioactive compounds and antioxidant activity of the pulp of the tropical fruit açai (*Euterpe oleracea* Mart.). Heliyon.

[52] Kyriakoudi A., Tsimidou M.Z. (2018). Properties of encapsulated saffron extracts in maltodextrin using the Büchi B-90 nano spray-dryer. Food Chem..

[53] Segneanu A.-E., Vlase G., Vlase T., Sicoe C.A., Ciocalteu M.V., Herea D.D., Ghirlea O.-F., Grozescu I., Nanescu V. (2023). Wild-Grown Romanian *Helleborus purpurascens* Approach to Novel Chitosan Phyto-Nanocarriers—Metabolite Profile and Antioxidant Properties. Plants.

[54] Vlase G., Segneanu A.-E., Bejenaru L.E., Bradu I.A., Sicoe C., Vlase T., Mogoşanu G.D., Buema G., Herea D.-D., Ciocîlteu M.V. (2025). Wild-Grown Romanian *Eupatorium cannabinum*: Advancing Phyto-Nanocarriers via Maltodextrin Micro-Spray Encapsulation—Metabolite Profiling, Antioxidant, Antimicrobial, and Cytotoxicity Insights. Polymers.

[55] Segneanu A.-E., Vlase G., Vlase T., Bejenaru L.E., Mogoşanu G.D., Buema G., Herea D.-D., Ciocîlteu M.V., Bejenaru C. (2024). Insight into Romanian Wild-Grown *Heracleum sphondylium*: Development of a New Phytocarrier Based on Silver Nanoparticles with Antioxidant, Antimicrobial and Cytotoxicity Potential. Antibiotics.

[56] Segneanu A.E., Vlase G., Marin C.N., Vlase T., Sicoe C., Herea D.D., Ciocîlteu M.V., Bejenaru L.E., Minuti A.E., Zară C.M. (2025). Wild grown *Portulaca oleracea* as a novel magnetite based carrier with in vitro antioxidant and cytotoxicity potential. Sci. Rep..

[57] Segneanu A.-E., Vlase G., Vlase T., Ciocalteu M.-V., Bejenaru C., Buema G., Bejenaru L.E., Boia E.R., Dumitru A., Boia S. (2024). Romanian Wild-Growing *Chelidonium majus*—An Emerging Approach to a Potential Antimicrobial Engineering Carrier System Based on AuNPs: In Vitro Investigation and Evaluation. Plants.

[58] Segneanu A.-E., Vlase G., Chirigiu L., Herea D.D., Pricop M.-A., Saracin P.-A., Tanasie Ş.E. (2023). Romanian Wild-Growing *Armoracia rusticana* L.—Untargeted Low-Molecular Metabolomic Approach to a Potential Antitumoral Phyto-Carrier System Based on Kaolinite. Antioxidants.

[59] Kavaz D., Faraj R.E.K.E. (2023). Investigation of composition, antioxidant, antimicrobial and cytotoxic characteristics from *Juniperus sabina* and *Ferula communis* extracts. Sci. Rep..

[60] Hossain M.L., Lim L.Y., Hammer K., Hettiarachchi D., Locher C. (2022). A Review of Commonly Used Methodologies for Assessing the Antibacterial Activity of Honey and Honey Products. Antibiotics.

[61] Andrews J.M. (2001). Determination of minimum inhibitory concentrations. J. Antimicrob. Chemother..

[62] Adil M., Filimban F.Z., Ambrin, Quddoos A., Sher A.A., Naseer M. (2024). Phytochemical screening, HPLC analysis, antimicrobial and antioxidant effect of *Euphorbia parviflora* L. (*Euphorbiaceae* Juss.). Sci. Rep..

[63] Kar S., Sengupta D., Deb M., Shilpi A., Parbin S., Rath S.K., Pradhan N., Rakshit M., Patra S.K. (2014). Expression profiling of DNA methylation-mediated epigenetic gene-silencing factors in breast cancer. Clin. Epigenetics.

[64] Babushok V.I., Zenkevich I.G. (2009). Retention Indices for Most Frequently Reported Essential Oil Compounds in GC. Chromatographia.

[65] Shareef H.K., Muhammed H.J., Hussein H.M., Hameed I.H. (2016). Antibacterial effect of ginger (*Zingiber officinale*) Roscoe and bioactive chemical analysis using gas chromatography mass spectrum. Orient. J. Chem..

[66] Nishidono Y., Saifudin A., Deevanhxay P., Tanaka K. (2020). Metabolite Profiling of Ginger (*Zingiber officinale* Roscoe) Using GC–MS and Multivariate Statistical Analysis. J. Asia Jpn. Res. Inst. Ritsumeikan Univ..

[67] Zhengkui L., Yingfang H. (1987). Chemical constituents of the essential oil from *Zingiber officinale* rose of Sichuan. Chin. J. Org. Chem..

[68] Edo G.I., Igbuku U.A., Makia R.S., Isoje E.F., Gaaz T.S., Yousif E., Jikah A.N., Zainulabdeen K., Akpoghelie P.O., Opiti R.A. (2025). Phytochemical profile, therapeutic potentials, nutritional composition, and food applications of ginger: A comprehensive review. Discov. Food.

[69] Styawan A.A., Susidarti R.A., Purwanto, Windarsih A., Rahmawati N., Sholikhah I.K.M., Rohman A. (2022). Review on ginger (*Zingiber officinale* Roscoe): Phytochemical composition, biological activities and authentication analysis. Food Res..

[70] Oforma C.C., Udourioh G.A., Ojinnaka C.M. (2019). Characterization of Essential Oils and Fatty Acids Composition of Stored Ginger (*Zingiber officinale* Roscoe). J. Appl. Sci. Environ. Manag..

[71] Zhang S., Zhang L., Yu M., Luo D., Chen S., Liu W., Zhang Y., Zhang L., Zhao T. (2022). Essential oils of *Zingiber officinale*: Chemical composition, *in vivo* alleviation effects on TPA induced ear swelling in mice and in vitro bioactivities. Front. Nutr..

[72] Wibowo D.P., Mariani R., Hasanah S.U., Aulifa D.L. (2020). Chemical Constituents, Antibacterial Activity and Mode of Action of Elephant Ginger (*Zingiber officinale* var. *officinale*) and Emprit Ginger Rhizome (*Zingiber officinale* var. *amarum*) Essential Oils. Pharmacogn. J..

[73] Sova M., Saso L. (2020). Natural Sources, Pharmacokinetics, Biological Activities and Health Benefits of Hydroxycinnamic Acids and Their Metabolites. Nutrients.

[74] Król M., Minkiewicz J., Mozgawa W. (2016). IR spectroscopy studies of zeolites in geopolymeric materials derived from kaolinite. J. Mol. Struct..

[75] Vargas-Muñoz D.P., Kurozawa L.E. (2020). Influence of combined hydrolyzed collagen and maltodextrin as carrier agents in spray drying of cocona pulp. Braz. J. Food Technol..

[76] Simon-Brown K., Mis Solval K., Chotiko A., Alfaro L., Reyes V., Liu C., Dzandu B., Kyereh E., Goldson Barnaby A., Thompson I. (2016). Microencapsulation of ginger (*Zingiber officinale*) extract by spray drying technology. LWT.

[77] Segneanu A.-E., Marin C.N., Herea D.D., Stanusoiu I., Muntean C., Grozescu I. (2022). Romanian *Viscum album* L.—Untargeted Low-Molecular Metabolomic Approach to Engineered *Viscum*–AuNPs Carrier Assembly. Plants.

[78] Sachan A., Penumadu D. (2007). Identification of Microfabric of Kaolinite Clay Mineral Using X-ray Diffraction Technique. Geotech. Geol. Eng..

[79] Edraki M., Moghaddampour I.M., Alinia-Ahandani E., Keivani M.B., Sheydaei M. (2021). Ginger intercalated sodium montmorillonite nano clay: Assembly, characterization, and investigation antimicrobial properties. Chem. Rev. Lett..

[80] Silva S., Alves N., Silva P., Vieira T., Maciel P., Castellano L.R., Bonan P., Velozo C., Albuquerque D. (2019). Antibacterial Activity of *Rosmarinus officinalis*, *Zingiber officinale*, *Citrus aurantium bergamia*, and *Copaifera officinalis* Alone and in Combination with Calcium Hydroxide against *Enterococcus faecalis*. Biomed Res. Int..

[81] Ferreira A.S., Silva A.M., Laveriano-Santos E.P., Lozano-Castellón J., Lamuela-Raventós R.M., Švarc-Gajíc J., Delerue-Matos C., Estevinho B.N., Costa P.C., Rodrigues F. (2024). Development and characterization of spray-drying microparticles loaded with chestnut shells extract: New insights for oral mucositis therapy. Powder Technol..

[82] Johnson J.B., Batley R.J., Mani J.S., Naiker M. (2023). How Low Can It Go? ATR–FTIR Characterization of Compounds Isolated from Ginger at the Nanogram Level. Eng. Proc..

[83] Karthickeyan V. (2018). Effect of nature based antioxidant from *Zingiber officinale* Rosc. on the oxidation stability, engine performance and emission characteristics with neem oil methyl ester. Heat Mass Transf..

[84] Alzarieni K.Z., Bani Amer A.R., El-Elimat T., Kenttämaa H.I. (2023). Characterization of Natural Cellulosic Fiber Obtained from the Flower Heads of Milk Thistle (*Silybum marianum*) as a Potential Polymer Reinforcement Material. J. Nat. Fibers.

[85] Dash D.K., Tyagi C.K., Sahu A.K., Tripathi V., Meena V.S., Parewa H.P., Meena S.K. (2022). Revisiting the medicinal value of terpenes and terpenoids. Revisiting Plant Biostimulants.

[86] Lieu E.L., Nguyen T., Rhyne S., Kim J. (2020). Amino acids in cancer. Exp. Mol. Med..

[87] Albaugh V.L., Pinzon-Guzman C., Barbul A. (2017). Arginine—Dual roles as an onconutrient and immunonutrient. J. Surg. Oncol..

[88] Kim S.H., Roszik J., Grimm E.A., Ekmekcioglu S. (2018). Impact of L-Arginine Metabolism on Immune Response and Anticancer Immunotherapy. Front. Oncol..

[89] Chiangjong W., Chutipongtanate S., Hongeng S. (2020). Anticancer peptide: Physicochemical property, functional aspect and trend in clinical application (Review). Int. J. Oncol..

[90] Liga S., Paul C., Péter F. (2023). Flavonoids: Overview of Biosynthesis, Biological Activity, and Current Extraction Techniques. Plants.

[91] Coniglio S., Shumskaya M., Vassiliou E. (2023). Unsaturated Fatty Acids and Their Immunomodulatory Properties. Biology.

[92] Vezza T., Canet F., de Marañón A.M., Bañuls C., Rocha M., Víctor V.M. (2020). Phytosterols: Nutritional Health Players in the Management of Obesity and Its Related Disorders. Antioxidants.

[93] Zhang Y., Cai P., Cheng G., Zhang Y. (2022). A Brief Review of Phenolic Compounds Identified from Plants: Their Extraction, Analysis, and Biological Activity. Nat. Prod. Commun..

[94] Lobiuc A., Pavăl N.-E., Mangalagiu I.I., Gheorghiţă R., Teliban G.-C., Amăriucăi-Mantu D., Stoleru V. (2023). Future Antimicrobials: Natural and Functionalized Phenolics. Molecules.

[95] Sun D.J., Zhu L.J., Zhao Y.Q., Zhen Y.Q., Zhang L., Lin C.C., Chen L.X. (2020). Diarylheptanoid: A privileged structure in drug discovery. Fitoterapia.

[96] Yu T.-J., Tang J.-Y., Shiau J.-P., Hou M.-F., Yen C.-H., Ou-Yang F., Chen C.-Y., Chang H.-W. (2022). Gingerenone A Induces Antiproliferation and Senescence of Breast Cancer Cells. Antioxidants.

[97] Huang Y., Cao S., Zhang Q., Zhang H., Fan Y., Qiu F., Kang N. (2018). Biological and pharmacological effects of hexahydrocurcumin, a metabolite of curcumin. Arch. Biochem. Biophys..

[98] Polaquini C.R., Morão L.G., Nazaré A.C., Torrezan G.S., Dilarri G., Cavalca L.B., Campos D.L., Silva I.C., Pereira J.A., Scheffers D.J. (2019). Antibacterial activity of 3,3’-dihydroxycurcumin (DHC) is associated with membrane perturbation. Bioorg. Chem..

[99] Xi H., Weng Y., Zheng Y., Wu L., Han D. (2024). Diacetoxy-6-gingerdiol protects the extracellular matrix of nucleus pulposus cells and ameliorates intervertebral disc degeneration by inhibiting the IL-1β-mediated NLRP3 pathway. Heliyon.

[100] Alberti Á., Riethmüller E., Béni S. (2018). Characterization of diarylheptanoids: An emerging class of bioactive natural products. J. Pharm. Biomed. Anal..

[101] Rahmani A.H., Al Shabrmi F.M., Aly S.M. (2014). Active ingredients of ginger as potential candidates in the prevention and treatment of diseases via modulation of biological activities. Int. J. Physiol. Pathophysiol. Pharmacol..

[102] Imm J., Zhang G., Chan L.Y., Nitteranon V., Parkin K.L. (2010). [6]-Dehydroshogaol, a minor component in ginger rhizome, exhibits quinone reductase inducing and anti-inflammatory activities that rival those of curcumin. Food Res. Int..

[103] Li F., Nitteranon V., Tang X., Liang J., Zhang G., Parkin K.L., Hu Q. (2012). In vitro antioxidant and anti-inflammatory activities of 1-dehydro-[6]-gingerdione, 6-shogaol, 6-dehydroshogaol and hexahydrocurcumin. Food Chem..

[104] Pandey V.K., Srivastava S., Ashish, Dash K.K., Singh R., Dar A.H., Singh T., Farooqui A., Shaikh A.M., Kovacs B. (2024). Bioactive properties of clove (*Syzygium aromaticum*) essential oil nanoemulsion: A comprehensive review. Heliyon.

[105] Hamdy A.E., Abdel-Aziz H.F., El-khamissi H., AlJwaizea N.I., El-Yazied A.A., Selim S., Tawfik M.M., AlHarbi K., Ali M.S.M., Elkelish A. (2022). Kaolin Improves Photosynthetic Pigments, and Antioxidant Content, and Decreases Sunburn of Mangoes: Field Study. Agronomy.

[106] Najem R.S. (2015). Antimicrobial activity of ginger (*Zingiber officinale*) extracts against *Klebsiella pneumoniae* and *Pseudomonas aeruginosa*. J. Biol. Nat..

[107] Dharmapala K.P., Amarakoon R. (2024). An Evaluation of Antimicrobial Activity of Common *Zingiber officinale* Cultivars Grown in Sri Lanka. Eur. J. Agric. Food Sci..

[108] Sebiomo A., Awofodu A.D., Awosanya A.O., Awotona F.E., Ajayi A.J. (2011). Comparative studies of antibacterial effect of some antibiotics and ginger (*Zingiber officinale*) on two pathogenic bacteria. J. Microbiol. Antimicrob..

[109] Shehu Z., Lamayi D.W., Sabo M.A., Shafiu M.M. (2018). Synthesis, Characterization and Antibacterial Activity of Kaolin/Gum Arabic Nanocomposite on *Escherichia coli* and *Pseudomonas aeruginosa*. Res. J. Nanosci. Eng..

[110] Isichei-ukah O.B., Ofie O.G. (2023). Antimicrobial Activity of Clay against Some Skin Infection Isolates. Niger J. Microbiol..

[111] Karaubayeva A.A., Bekezhanova T., Zhaparkulova K., Susniak K., Sobczynski J., Kazimierczak P., Przekora A., Skalicka-Wozniak K., Kulinowski Ł., Glowniak-Lipa A. (2024). Antimicrobial Mixture Based on Micronized Kaolinite and *Ziziphora* Essential Oil as a Promising Formulation for the Management of Infected Wounds. Int. J. Mol. Sci..

[112] Radhakrishnan E.K., Bava S.V., Narayanan S.S., Nath L.R., Thulasidasan A.K.T., Soniya E.V., Anto R.J. (2014). [6]-Gingerol Induces Caspase-Dependent Apoptosis and Prevents PMA-Induced Proliferation in Colon Cancer Cells by Inhibiting MAPK/AP-1 Signaling. PLoS ONE.

[113] Zivarpour P., Nikkhah E., Maleki Dana P., Asemi Z., Hallajzadeh J. (2021). Molecular and biological functions of gingerol as a natural effective therapeutic drug for cervical cancer. J. Ovarian Res..

[114] Choi N.R., Choi W.G., Kwon M.J., Woo J.H., Kim B.J. (2022). [6]-Gingerol Induces Caspase-Dependent Apoptosis in Bladder Cancer Cells via MAPK and ROS Signaling. Int. J. Med. Sci..

[115] Sarami S., Dadmanesh M., Hassan Z.M., Ghorban K. (2020). Study on the Effect of Ethanol Ginger Extract on Cell Viability and p53 Level in Breast and Pancreatic Cancer. Arch. Pharm. Pract..

[116] Malmir S., Ebrahimi A., Mahjoubi F. (2020). Effect of ginger extracts on colorectal cancer HCT-116 cell line in the expression of MMP-2 and KRAS. Gene Rep..

[117] Yao G.L., Li H., Teng L., Fan Y., Huang W. (2024). Green decorated of gold nanoparticles over kaolin mediated by plant extract and investigation of its anti-bile duct cancer properties in the in vitro condition. Arab. J. Chem..

[118] Gianni E., Avgoustakis K., Papoulis D. (2020). Kaolinite group minerals: Applications in cancer diagnosis and treatment. Eur. J. Pharm. Biopharm..

[119] Alqahtani M.D., Nasser N., AlZahrani S.A., Allam A.A., Abukhadra M.R. (2023). Characterization of Kaolinite Single Methoxy Nano-Sheets as Potential Carriers of Oxaliplatin Drug of Enhanced Loading, Release, and Cytotoxicity Properties During the Treatment of Colorectal Cancer. J. Inorg. Organomet. Polym. Mater..

[120] Tian L., Abukhadra M.R., Mohamed A.S., Nadeem A., Ahmad S.F., Ibrahim K.E. (2020). Insight into the Loading and Release Properties of an Exfoliated Kaolinite/Cellulose Fiber (EXK/CF) Composite as a Carrier for Oxaliplatin Drug: Cytotoxicity and Release Kinetics. ACS Omega.

[121] Rozhina E., Batasheva S., Miftakhova R., Yan X., Vikulina A., Volodkin D., Fakhrullin R. (2021). Comparative cytotoxicity of kaolinite, halloysite, multiwalled carbon nanotubes and graphene oxide. Appl. Clay Sci..

[122] Segneanu A.-E., Vlase G., Vlase T., Bita A., Bejenaru C., Buema G., Bejenaru L.E., Dumitru A., Boia E.R. (2024). An Innovative Approach to a Potential Neuroprotective *Sideritis scardica*–Clinoptilolite Phyto-Nanocarrier: In Vitro Investigation and Evaluation. Int. J. Mol. Sci..

[123] Salem M.A.I., Marzouk M.I., El-Kazak A.M. (2016). Synthesis and Characterization of Some New Coumarins with In Vitro Antitumor and Antioxidant Activity and High Protective Effects against DNA Damage. Molecules.

[124] Al Hafiz S.S., Syofyan S., Alen Y., Hamidi D. (2024). FT–IR Fingerprinting Analysis for Classification of West Sumatra Small Ginger (*Zingiber officinale* Roscoe) Essential Oil and its Antioxidant Activity. Trop. J. Nat. Prod. Res..

[125] Gaikwad D.D., Shinde Sachin K., Kawade Ashwini V., Jadhav S.J., Gadhave M.V. (2017). Isolation and standardization of gingerol from ginger rhizome by using TLC, HPLC, and identification tests. Pharma. Innov. J..

[126] Singh V., Pandey H., Misra V., Singh D. (2022). Biocompatible herbal polymeric nano-formulation of [6]-gingerol: Development, optimisation, and characterization. Ecol. Environ. Conserv..

[127] Scarsini M., Thurotte A., Veidl B., Amiard F., Niepceron F., Badawi M., Lagarde F., Schoefs B., Marchand J. (2021). Metabolite Quantification by Fourier Transform Infrared Spectroscopy in Diatoms: Proof of Concept on *Phaeodactylum tricornutum*. Front. Plant Sci..

